# Integrative analyses and validation of ferroptosis-related genes and mechanisms associated with cerebrovascular and cardiovascular ischemic diseases

**DOI:** 10.1186/s12864-023-09829-w

**Published:** 2023-12-04

**Authors:** Wei Liao, Yuehui Wen, Chuan Zeng, Shaochun Yang, Yanyu Duan, Chunming He, Ziyou Liu

**Affiliations:** 1 Department of Neurosurgery, First Affiliated of Gannan Medical University, Ganzhou, Jiangxi China; 2 Key Laboratory of Prevention and Treatment of Cardiovascular and Cerebrovascular Diseases, Ministry of Education, Gannan Medical University, Ganzhou, Jiangxi China; 3 Shanghai Jiao Tong University School of Medicine, Shanghai, China; 4 Gannan Medical University, Ganzhou, Jiangxi China; 5 Heart Medical Centre, First Affiliated of Gannan Medical University, Ganzhou, Jiangxi China; 6 Department of Cardiac Surgery, First Affiliated of Gannan Medical University, Ganzhou, Jiangxi China

**Keywords:** Ferroptosis, Cerebrovascular ischemic diseases, Cardiovascular ischemic diseases, Machine algorithms, Common pathway of injury, Diagnostic marker

## Abstract

**Background:**

There has been a gradual increase in the occurrence of cardiovascular and cerebrovascular ischemic diseases, particularly as comorbidities. Yet, the mechanisms underlying these diseases remain unclear. Ferroptosis has emerged as a potential contributor to cardio-cerebral ischemic processes. Therefore, this study investigated the shared biological mechanisms between the two processes, as well as the role of ferroptosis genes in cardio-cerebral ischemic damage, by constructing co-expression modules for myocardial ischemia (MI) and ischemic stroke (IS) and a network of protein–protein interactions, mRNA-miRNA, mRNA-transcription factors (TFs), mRNA-RNA-binding proteins (RBPs), and mRNA-drug interactions.

**Results:**

The study identified seven key genes, specifically ACSL1, TLR4, ADIPOR1, G0S2, PDK4, HP, PTGS2, and subjected them to functional enrichment analysis during ischemia. The predicted miRNAs were found to interact with 35 hub genes, and interactions were observed between 11 hub genes and 30 TF transcription factors. Additionally, 10 RBPs corresponding to 16 hub genes and 163 molecular compounds corresponding to 30 hub genes were identified. This study also clarified the levels of immune infiltration between MI and IS and different subtypes. Finally, we identified four hub genes, including TLR4, by using a diagnostic model constructed by Least Absolute Shrinkage and Selection Operator (LASSO) regression analysis; ADIPOR1, G0S2, and HP were shown to have diagnostic value for the co-pathogenesis of MI and cerebral ischemia by both validation test data and RT-qPCR assay.

**Conclusions:**

To the best our knowledge, this study is the first to utilize multiple algorithms to comprehensively analyze the biological processes of MI and IS from various perspectives. The four hub genes, TLR4, ADIPOR1, G0S2, and HP, have proven valuable in offering insights for the investigation of shared injury pathways in cardio-cerebral injuries. Therefore, these genes may serve as diagnostic markers for cardio-cerebral ischemic diseases.

**Supplementary Information:**

The online version contains supplementary material available at 10.1186/s12864-023-09829-w.

## Introduction

The prevalence of cardio-cerebral comorbidities has been significantly increasing. Historically, most studies and therapies for cardio-cerebral vascular diseases have focused solely on a singular disease within the cardio-cerebral vascular system. This approach fails to provide a comprehensive and integrated diagnosis and treatment plan for patients with cardio-cerebral comorbidities, thereby impeding the precise management of cardio-cerebral vascular comorbidities. Research indicates that the mechanisms of injury in myocardial and cerebral ischemia are similar. Both conditions share a common pathological mechanism known as atherosclerosis [1]. Additionally, the pathological state following the onset of cardio-cerebral ischemic disease is closely associated with ischemia–reperfusion injury [2]. Furthermore, many molecular events that occur after neurological injury also occur after cardiac injury [3]. These findings suggest the presence of a shared injury mechanism in the heart and brain. Nevertheless, the precise cause of the injury remains uncertain and requires further investigation. Therefore, exploring shared therapeutic objectives for heart-brain injury, administering targeted treatments to safeguard the heart and brain, and adopting a brain-centered homeopathic approach, is imperative.

Our team has shown that glutamate receptors serve as prevalent indicators of damage in cardiac and cerebral ischemia-hypoxia-reperfusion injuries. Additionally, it can control the progression of cardiac and cerebral ischemia/reperfusion injury [4–6]. However, further studies are required to elucidate the underlying mechanisms. Research indicates that during cardiac transplantation and coronary artery occlusion, cardiomyocytes undergo ferroptosis, leading to inflammation and worsening of cardiac injury. The utilization of Fer-1, a ferroptosis inhibitor, has been shown to decrease the occurrence of ferroptosis in cardiomyocytes [7]. Additionally, in mice undergoing surgery for middle cerebral artery occlusion, ferroptosis intensifies surgically induced cerebral ischemia/reperfusion injury [8].

A recent biological information and validation study on ferroptosis-related genes (FRGs) during ischemic stroke (IS) screened hub genes related to ferroptosis and suggested a possible mechanism for dexmedetomidine-mediated inhibition of ferroptosis during IS [9], and during myocardial ischemia (MI), the same biosignature study identified genes related to ferroptosis and carried out immune infiltration analyses and confirmation of related hub genes [10, 11], these studies separately from the direction of MI and IS to explore the role of FRGs in MI and IS, but did not further study the common characteristics of the process of cardio-cerebral ischemia, and to further clarify the process of cardio-cerebral ischemia with a consistent trend of expression of the hub genes of ferroptosis, which is precisely the problem explored in this paper, the study of the FRGs in cardio-cerebral ischemia. The study of the common role of ferroptosis genes in ischemia is of great significance in the search for common pathways and mechanisms of ischemic injury.

This study aimed to investigate the role of ferroptosis in cardiac and cerebral ischemic injuries by exploring the common pathways of injury between the two organs. Using various machine algorithms, the optimal model of the most relevant FRGs in heart and brain injury was determined. Bioinformatics, clinical specimens, and cell model validation were used to study the hub genes and biological processes associated with ferroptosis during MI and IS. This analysis aimed to identify common injury mechanisms and targets for cardio-cerebral ischemia–reperfusion injury, which can aid in early intervention and treatment.

## Materials and methods

### Data acquisition

The MI related datasets GSE60993 [12], GSE66360 [13], and GSE48060obtained from the GEO database (https://www.ncbi.nlm.nih.gov/geo) [14]. The GSE60993 dataset was acquired from *Homo sapiens*, using the data platform being GPL6884. It consisted of 33 samples, including 17 MI, 7 normal, and 9 unstable angina pectoris samples. For the present analysis, only 17 MI samples and 7 normal samples from GSE60993 were included. TheGSE66360 dataset was obtained from Homo sapiens, with the data platform being GPL570. It comprises 99 samples, including 49 MI samples and 50 normal samples. All the samples were selected for analysis. Finally, the GSE48060 dataset, also from *Homo sapiens*, used the GPL570 data platform and contained 52 samples, including 31 MI and 31 normal samples. This analysis included 31 samples, including 21 normal samples. The training datasets used were GSE60993 and GSE66360, while GSE48060 was used as the validation dataset. Additionally, the GEO database provided IS associated datasets GSE22255 [15], GSE16561 [16], and GSE58294, which were downloaded. Datasets GSE22255, which consisted of 40 samples (20 IS cases and 20 normal cases), and GSE16561, which consisted of 63 samples (39 IS cases and 24 normal cases), were included in the analysis. The dataset GSE58294, which consisted of 92 samples (69 IS cases and 23 normal cases), was used as the validation dataset. All the samples were selected for inclusion in the analysis. The data platforms used were GPL570 for GSE22255 and GSE58294, and GPL6883 for GSE16561. Specific grouping information for the datasets can be found in the Supplementary material (Additional file 1: Table S1).

The GeneCards database (https://www.genecards.org) [17] was used to identify genes associated with ferroptosis. Supplementary Material (Additional file 2: Table S2) displays the specific names of the 698 genes acquired from the GeneCards database using the search term “Ferroptosis”.

### Identification of differentially expressed genes (DEGs) associated with ferroptosis

To obtain the integrated MI dataset, we used the R package sva to de-batch the datasets GSE60993 and GSE66360, comprising 66 MI cases and 57 control (normal) samples. Additionally, we obtained an integrated IS dataset using datasets GSE22255 and GSE16561, which included 59 IS and 44 control (normal) samples. After normalizing the combined dataset using the limma package [18], the effectiveness of batch effect removal was confirmed by conducting Principal Component Analysis (PCA) on the expression matrix of the dataset both before and after the batch effect was eliminated. PCA is a data dimensionality reduction method that extracts the feature vectors (components) of high-dimensional data, converts them to low-dimensional data and displays these features in a two-dimensional graph.

The expression data of genes related to ferroptosis in the MI and IS were acquired by intersecting the gene expression data of the FRGs with the respective data for the MI and IS. To examine the impact of FRG gene expression levels in MI and IS, we conducted differential gene analysis on the combined dataset. This analysis utilized the R package limma [19] to identify significant differential genes. The threshold for differential genes was set at a fold change (FC) absolute value > 1.5 and *p* < 0.05. Additionally, genes with FC > 1.5 and *p* < 0.05 were considered up-regulated in expression. Differential genes were defined as those with FC > 1.5 and *p* < 0.05. Up-regulated differential genes were identified as those with FC > 1.5 and *p* < 0.05, while down-regulated differential genes were identified as those with FC < -1.5 and *p* < 0.05. The expression patterns of genes related to iron-induced cell death were illustrated using the ggplot2 package in R for volcano plotting and the pheatmap package in R for heatmap visualization.

### Assessment of biological characteristics between disease and control samples

 Gene Ontology (GO) enrichment analysis is a widely used approach to study the functional enrichment of genes on a large scale, across various dimensions and levels. This analysis is typically conducted on three levels: biological process (BP), molecular function (MF), and cellular component (CC) [18]. Kyoto Encyclopedia of Genes and Genomes (KEGG) is an extensively utilized repository for housing data on genomes, biological pathways, diseases, and medications [20]. Disease Ontology (DO) provides gene annotations from a disease standpoint. The R package clusterProfiler [21, 22] was used to perform GO functional annotation, KEGG pathway enrichment, and DO disease enrichment in order to identify significantly enriched biological processes. This analysis was conducted on DEGs related to ferroptosis between the disease and control samples from the integrated dataset of MI and IS. The significance threshold for enrichment analysis was set at *p* < 0.05.

Gene Set Enrichment Analysis (GSEA) is a computational technique used to assesswhether a pre-established group of genes exhibits significant variances between two biological conditions. It is frequently employed to evaluate alterations in pathways and biological process activity within expression dataset samples [23]. In order to examine the disparities in biological mechanisms between MI and IS disease samples and control samples, we utilized gene expression profiling datasets and referred to the gene sets 'c5.go.v7.4.entrez.gmt' and 'c2.cp.kegg’. v7.4. The datasets from entrez.gmt [24] were enriched and visualized using the GSEA method provided in the R package clusterProfiler. *p* < 0.05 was considered statistically significant.

Gene Set Variation Analysis (GSVA) is an unsupervised analysis technique that is primarily employed to assess the gene expression matrix across various samples by converting it into a gene set expression matrix using a non-parametric approach. The enrichment results of the transcriptome gene sets were used to evaluate whether distinct metabolic pathways are enriched across various samples. To examine the variation in biological processes between disease and control samples of MI and IS in the combined dataset, we conducted gene set variation analysis on the gene expression profiling data of the disease and control samples of MI and IS. This analysis was performed using the R package” GSVA” [25], and the reference gene set was obtained from the MSigDB database [24]. The dataset's enrichment scores for each hallmark were calculated using the set 'h.all.v7.4.symbols.gmt', and the correlation between the dysregulated pathways in the patients was determined. *p* < 0.05 was considered statistically significant.

### Construction of a diagnostic model for Ferroptosis

A risk score formula was established using the Least absolute shrinkage and selection operator (LASSO) algorithm analysis to reduce dimensionality and identify differentially expressed feature genes among the genes related to ferroptosis. The formula incorporates the individual normalized gene expression values weighted by the penalty coefficients of the feature genes.

$$\mathrm{riskScore}=\sum_{\mathrm i}\mathrm{Coefficient}\left({\mathrm{gene}}_{\mathrm i}\right)\ast\mathrm{mRNA}\;\mathrm{Expression}({\mathrm{gene}}_{\mathrm i})$$

Random Forest (RF) [26] is a combination of multiple algorithms that integrates multiple decision trees using the concept of integration learning. This belongs to the bagging (Bootstrap AGGregatING, Self-Service Sampling Integration) integration algorithm among other integration algorithms. The random forest is a frequently used technique for constructing models. This involves creating multiple decision trees and using the statistics of each tree to predict a specific sample. The final result was determined by selecting the most common prediction among the trees using a voting method. The RF package [27] was used for model construction using the expression matrix of DEGs related to ferroptosis in the integrated dataset. The parameters set.seed(234) and ntree = 1000 were employed.

$$I(X=xi)= -lo{g}_{2}p({x}_{i})$$

An Support Vector Machine (SVM) is a generalized linear classifier used in supervised learning to classify data into two categories. It determines the decision boundary as a hyperplane with a maximum margin, which is obtained by solving the learning samples. SVM calculate the practical risk by a hinge loss function and incorporates a regularization term into the solution system to optimize the structural risk. Kernel methods enable SVM to perform nonlinear classifications, making them sparse and robust classifiers. SVM employs a hinge loss function to calculate the empirical risk and incorporates a regularization term to optimize the structural risk, making it a classifier that is both sparse and resilient. The kernel method allows the SVM to perform nonlinear classification, making it a popular choice among kernel learning methods.

The Receiver Operating Characteristic (ROC) [28] is a graphical analysis tool that enables the selection of the most suitable model, elimination of the second-best model, or determination of the optimal threshold within a single model. The ROC curveis a combined measure that represents the sensitivity and specificity. This illustrated the interplay between sensitivity and specificity using a compositional approach. Typically, the area under the ROC curve ranges from 0.5—1. The diagnostic performance improved as the area under the curve (AUC) approaches 1. The AUC exhibits low accuracy ranging from 0.5 to 0.7, moderate accuracy from 0.7 to 0.9, and high accuracy at 0.9 and beyond.

To identify the diagnostic indicators linked to ferroptosis in MI and IS, the integrated datasets of MI and IS were subjected dimensionality reduction using there techniques: LASSO, RF, and SVM. Subsequently, ROC Curves were generated using the R package pROC [29], and the AUC was calculated to evaluate the precision of the diagnostic models and determine the most suitable model.

### Weighted Gene Association Co-expression Network Analysis (WGCNA)

The WGCNA algorithm was used to examine the gene expression patterns in numerous samples to, facilitate gene clustering and module creation based on comparable gene expression patterns. Additionally, it allows for the analysis of connections between modules and their biological characteristics [30]. For this investigation, we employed the R software WGCNA [30] to construct gene co-expression networks pertaining to MI and IS. Initially, we utilized the ideal soft threshold β (8 for MI and 7 for IS) to create scale-free networks, individually. Next, the dissimilarity Topology Overlap Matrix (TOM)-based dissimilarity (dissTOM) was calculated and used to perform gene dendrograms and module identification with a maxClusterSize of 6000 and a minClusterSize of 30 to perform hierarchical clustering to identify co-expression modules. Subsequently, we computed Pearson’s correlation Coefficient (PCC) and its corresponding p value. These results were visualized using a heatmap to compare the module feature genes with clinical features. From this analysis, we identified disease-associated genes by selecting gene modules with an absolute correlation value greater than 0.3 and *p* < 0.05, which were considered clinically relevant.

### Building a network of protein-protein interaction (PPI), mRNA-miRNA, mRNA-RBP, mRNA-TF and mRNA-drugs interactions

The PPI network [31] consists of proteins that interact with each other to engage in different aspects of life processes, including biological signaling, gene expression regulation, energy and material metabolism, and cell cycle control. The Search Tool for the Retrieval of Interacting Genes/Proteins (STRING) database [32] serves as a repository for exploring identified proteins and forecasting protein interactions. In this study, we utilized the STRING database to link the co-morbid genes of MI and IS with a PPI network (minimum required interaction score low confidence [0.150]. Subsequently, we exported the PPI and employed Cytoscape [33] software for additional analysis. The Cytohubba [34] plug-in consists of 12 algorithms (Betweennes, BottleNeck, Closeness, ClusteringCoefficien, Degre, DMN, EcCentricity, EPC, MCC, MNC, Radiality, and Stress), which calculate the top 30 nodes in each algorithm. Genes that appeared in at least 5 algorithms were defined as hub nodes, indicating their significant connectivity with other nodes. These hub nodes may play crucial roles in regulating the entire biological process and warrant further investigation.

MiRNAs, which are single-stranded RNA molecules encoded by endogenous genes, are a type of non-coding RNA with a length of approximately 19–25 nt. They play crucial regulatory roles in the evolution of biological development. In post-transcriptional gene regulation, miRNAs play crucial roles in tumorigenesis, biological development, organogenesis, and epigenetic regulation by effectively controlling target gene expression. Viral defense mechanisms play important regulatory roles. Typically, miRNAs possess an intricate regulatory system, in which a single miRNA can control numerous target genes; conversely, a target gene can be regulated by multiple miRNAs [27]. To examine the correlation between hub genes and miRNAs, we acquired miRNAs associated with hub genes from the Starbase database (http://starbase.sysu.edu.cn/). This database offers seven prediction programs (TargetScan, microT, miRmap, PicTar, RNA22, PITA, and miRanda) for predicting the results. To construct the mRNA-miRNA regulatory network, interactions between miRNAs and mRNAs were predicted using two or more programs,and Cytoscape software were used to visualize the mRNA-miRNA regulatory network.

Transcription factors (TFs) through gene expression control. To examine the regulatory function of central genes, the miRNet database was used to retrieve and construct the interaction network between TFs and central genes. Using the Cytoscape software, we visualized the network of interactions between hub genes and TFs.

Furthermore, the Encyclopedia of RNA Interactomes (ENCORI) database was used to predict the interactions between RBP and hub genes. We then filtered the mRNA-RBP interaction pairs based on the criteria of clipExpNum >  = 5 and clipIDnum > 10, and subsequently constructed the mRNA-RBP interaction network.

The Comparative Toxicogenomics Database (CTD) (http://ctdbase.org/) is a digital platform that connects chemicals, genes, phenotypes, and diseases, and establishes toxicological data to enhance the comprehension of human health-related information. By applying the screening criterion of having at least three references and two organisms, the CTD database was used to identify potential medications or small molecule compounds that interact with hub genes. Furthermore, we employed Cytoscape to create a visual representation of the network of interactions between mRNA and drugs.

### Molecular subtypes of Ferroptosis

The Uniform Manifold Approximation and Projection (UMAP) algorithm, a nonlinear dimensionality reduction technique, can partition or condense a group of individuals into a sequence of separate clusters using specified characteristics. By utilizing the umap package in R [35], the algorithm categorizes the integrated dataset of MI and IS patients into distinct subtypes, relying on characteristic genes.

### Exploration of immune infiltration

The immune microenvironment is composed of a complex interconnected network primarily comprising immune cells, cells involved in inflammation, fibroblasts, and mesenchymal cells, as well as a variety of cytokines and chemokines. The examination of immune cell infiltration in samples plays a crucial role in guiding disease research and predicting treatment prognosis. Single sample gene set enrichment analysisss (GSEA) algorithm extends the GSEA method to determine the levels of` 28 immune cells in disease and control samples [32]. This information was presented as box plots, allowing visualization of the immune cell composition in patients with various MI and IS subtypes. Statistical significance was determined using the Wilcoxon test to calculate variations in the proportions of immune cells. A *p*-value < 0.05 was considered statistically significant.

CIBERSORx utilizes a machine learning technique that expands the algorithm framework to deduce gene expression profiles specific to cell types, eliminating the requirement for physically isolating cells. RNA-Seq data [36] is utilized to approximate the quantity of immune cells present in a specimen. Using the CIBERSORTx algorithm (https://cibersortx.stanford.edu/), we determined the prevalence of 22 immune cell types in various patient subtypes within the integrated MI and IS datasets. The R package corrplot [37] was used to create heat maps showing the correlation between the degree of immune cell infiltration.

ESTIMATE analysis is a computational method that measures the level of immune infiltration in tumor samples using gene expression data that indicates the presence of stromal and immune cell gene signatures. The ESTIMATE package for R [38] was used to estimate variations in immune scores between control samples and patients with MI and IS. This package calculates the correlation between hub gene expression levels and immune scores.

### Modeling of cellular hypoxia-reoxygenation

Hypoxia-reoxygenation models were created using CL-0481 rat neuronal PC12 cells from ProCell Life Science&Technology Co., Ltd. (Wuhan, China) and iCell-r012 rat cardiomyocyte H9c2 cells from iCell Bioscience Co., Ltd. (Shanghai, China). The model was validated using a CCK-8 assay and flow cytometry., The detailed procedure has been described elsewhere [4], and the cells were gathered and preserved in a − 80℃ facility.

### Clinical blood specimen collection

To further identify the presence of hub genes in brain and heart ischemia, we obtained nine blood samples from individuals undergoing clinical cardiopulmonary resuscitation, 12 blood samples from patients with acute IS, 12 blood pressure samples from patients with acute MI, and 12 blood pressure samples from individuals with a normal physical examination between March and October 2022. The collection time and specific treatments for all blood samples as described in our previous study [4].

### Validation using real-time fluorescence quantitative PCR (RT-qPCR)

RT-qPCR was used to determine the mRNA levels of hub genes. RNA was extracted using the CWBIO Ultrapure RNA Extraction Kit (CW0581M). The cDNA was synthesized using a reverse transcription kit (R223-01; Vazyme). The procedure involves three steps. Each reaction was a total volume of 20 μL, consisting of 10 μL ChamQ Universal SYBR qPCR Master Mix (Q711-02, Vazyme China) (2X), 4 μL of both forward and reverse primers, 1 μL cDNA, and the necessary amount of nuclease-free water. Each sample was analyzed in triplicate. The particular cycling conditions were as follows: initial denaturation at a temperature of 95 °C for 10 min, followed by denaturation at 95 °C for 10 s, annealing at 58 °C for 30 s, and extension at 72 °C for 30 s. A melting curve (final dissociation curve) was generated. The 2^−ΔΔCt^ method was used to calculate the relative expression of the gene, with β-actin chosen as the endogenous reference. Supplementary Material (Additional file 3: Table S3 and Additional file 4: Table S4) displays the primers used.

### Statistical analysis

R software (version 4.1.1) was used for all data processing and analyses. The statistical significance of variables that followed a normal distribution was assessed using an independent t-test to compare the two groups of continuous variables. Differences between variables that did not follow a normal distribution were analyzed using the Wilcoxon rank-sum test to compare the two groups of independent variables. Pearson’s correlation analysis was used to calculate correlation coefficients between various genes. ROC curves were generated using the R package pROC to evaluate the precision of the diagnostic model, and the AUC was computed. The statistical significance of all p-values was assessed using a two-sided test, and *p* < 0.05 was considered to indicate statistical significance. The results of RT-qPCR are reported as means ± standard deviation. Comparison of two groups of data that were normally distributed was conducted using the Student’s t-test. Groups were compared using one-way analysis of variance (ANOVA). Unless otherwise specified, *p* value of < 0.05 was considered statistically significant.

## Results

### Analysis of DEGs associated with ferroptosis

**Fig. 1:**
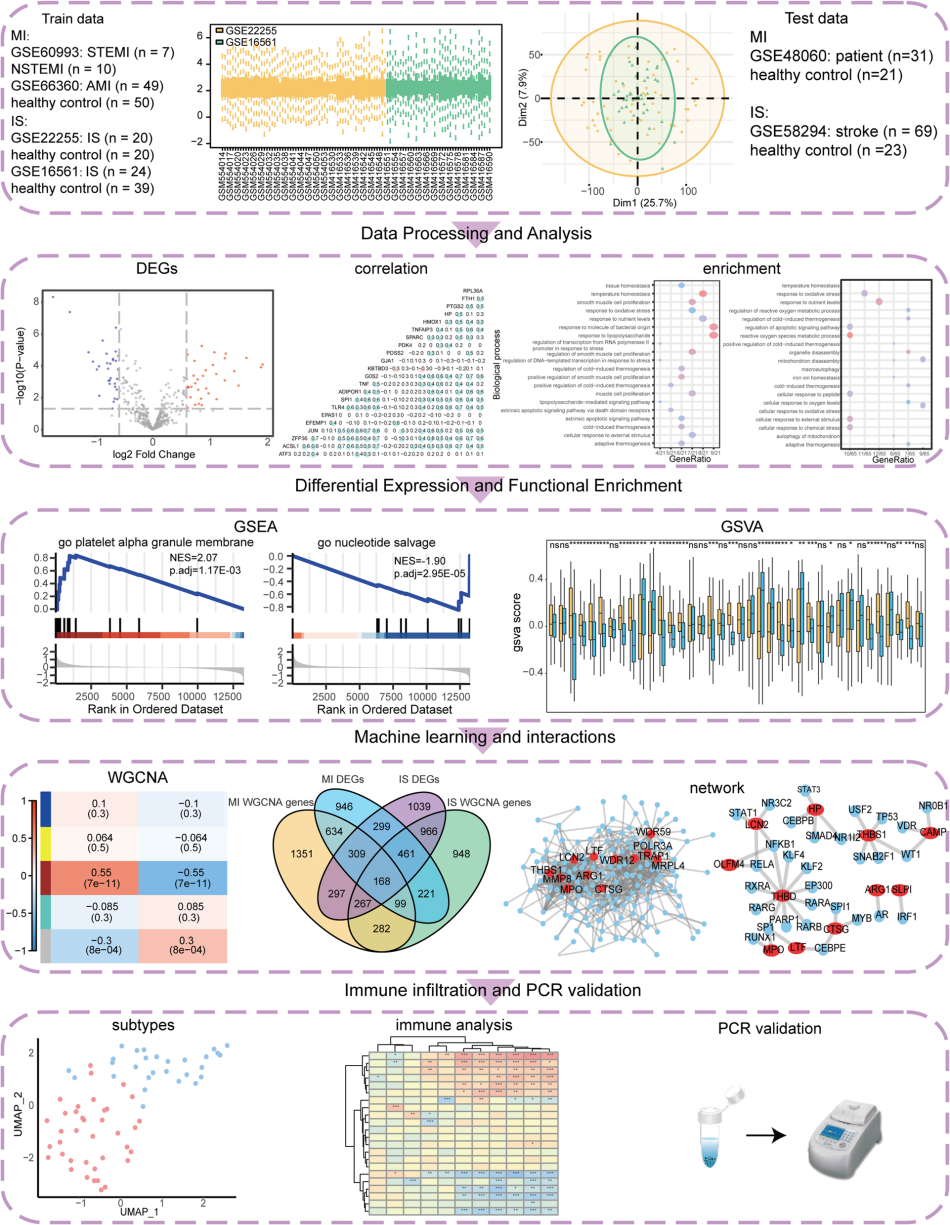
Technology flowchart. MI: Myocardial Infarction. IS: Ischemic Stroke

According to the flowchart (Fig. 1), the MI datasets GSE60993 and GSE66360 were initially combined (Additional file 5: Figure S1A), revealing a noticeable batch effect in the merged data (Additional file 5: Figure S1C). Subsequently, the batch effect was eliminated from the datasets (Additional file 5: Figure S1B) to obtain gene expression profile data with consistent expression levels (Additional file 5: Figure S1D). The integrated data comprised 66 MI and 57 normal samples. Subsequently, the IS datasets GSE22255 and GSE16561 were combined (Additional file 5: Figure S1E), which revealed a noticeable discrepancy between them (Additional file 5: Figure S1G). This discrepancy was eliminated from the datasets (Additional file 5: Figure S1F), resulting in gene expression profiling data that exhibited uniform expression levels (Additional file 5: Figure S1H). The integrated data comprised of data from 59 patients with IS and 44 healthy controls.

**Fig. 2:**
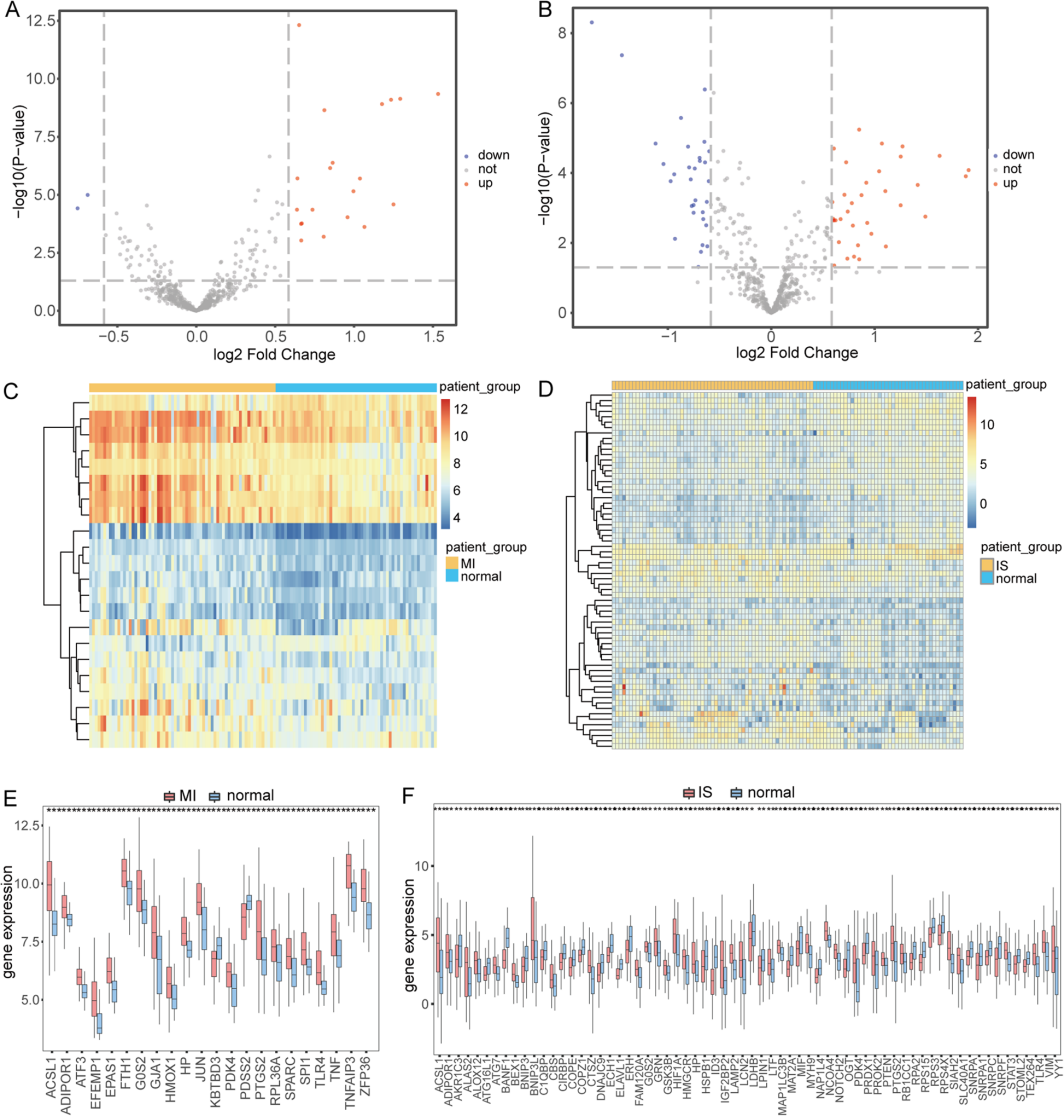
Examination of DEGs in relation to ferroptosis was conducted using combined MI and IS dataset. **A** Volcano plot of differentially expressed MI genes. Red nodes indicate upregulated genes, blue nodes indicate downregulated genes, and gray nodes indicate genes that do not differ significantly. **B** Volcano plot showing IS DEGs. **C** Heatmap displaying the expression levels of DEGs in MI, with MI samples represented in yellow and normal samples represented in blue. **D** Expression level heatmap displaying DEGs, with IS samples represented in yellow and normal samples represented in blue. **E** Boxplot differentially expressed boxplots of ferroptosis-related MI DEGs in disease and normal samples. *p < 0.05; **p < 0.01; ***p < 0.001. **F** Boxplot of differential expression of ferroptosis-related IS DEGs in disease and normal samples. *p < 0.05; **p < 0.01; ***p < 0.001. MI: Myocardial Infarction. IS: Ischemic Stroke. DEGs: Differentially Expressed Genes

To identify the DEGs associated with ferroptosis, we obtained the expression data of FRGs in MI and IS by intersecting the combined datasets of MI and IS with the FRGs. The analysis resulted in 22 DEGs, with 20 upregulated (FC > 1.5 and *p* < 0.05) and 2 downregulated genes (FC <  − 1.5 and *p* < 0.05) (Fig. 2A). The DEGs were visualized using the R package pheatmap (Fig. 2C), which revealed significant differences in expression among the different subgroups of the combined MI dataset. The analysis of IS and control samples resulted in the identification of 66 differentially expressed genes: 35 upregulated and 31 downregulated genes (Fig. 2B). Heat maps created using the R package pheatmap (Fig. 2D) clearly indicate the 66 differentially expressed genes in various subgroups of the combined IS dataset. Box plots of expression levels revealed that all genes related to ferroptosis that were differentially expressed in MI showed significant differential expression in both disease and normal samples (Fig. 2E). Similarly, all genes related to ferroptosis, which were differentially expressed in IS, also showed significant differential expression in both the disease and normal samples (Fig. 2F).

**Fig. 3:**
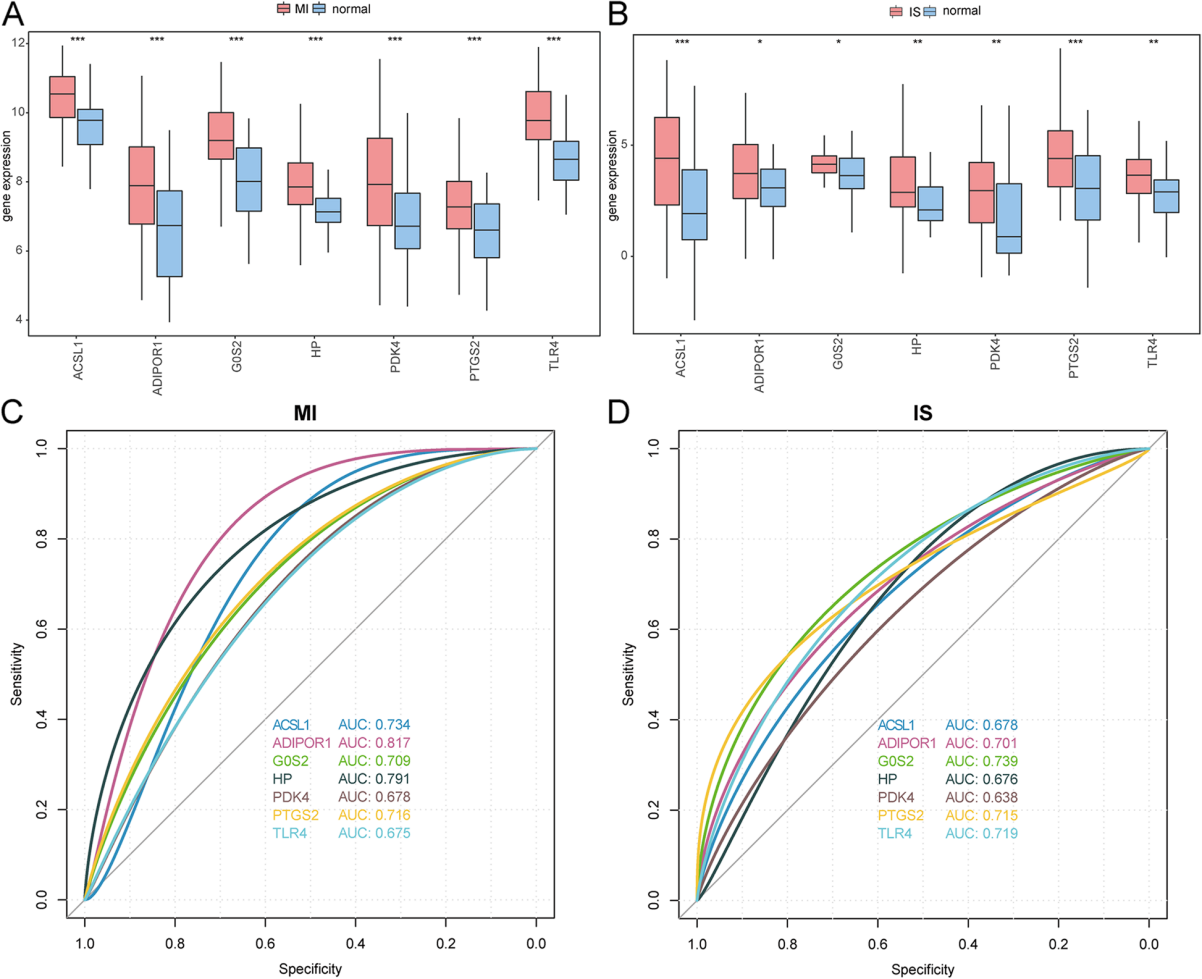
Analysis of genes related to ferroptosis that were co-differently expressed in MI and IS. **A** Boxplots presenting the differential expression of genes related to ferroptosis, which are co-differentially expressed in MI samples and normal samples, for MI and IS. **B** Differential expression box plots of genes related to ferroptosis, which are co-differentially expressed in MI and IS in IS samples compared to normal samples, can be visualized using boxplots. **C** ROC curves were generated for 7 DEGs related to ferroptosis in the combined MI dataset, including MI and IS. **D** ROC curves were generated for 7 FRGs that were differentially expressed in both MI and IS. These genes were identified in the combined MI dataset. *p < 0.05; **p < 0.01; ***p < 0.001. MI: Myocardial Infarction. IS: Ischemic Stroke. ROC: Receiver Operarating Curve. FRGs: Ferroptosis-related Genes. DEGs: Differentially Expressed Genes

To identify the FRGs that showed co-differential expression in both MI and IS, we intersected 22 differentially expressed FRGs from the merged MI dataset with 65 differentially expressed FRGs from the merged IS dataset. This resulted in the identification of seven FRGs (ACSL1, TLR4, ADIPOR1, G0S2, PDK4, HP, and PTGS2) that were differentially expressed in both MI and IS. Box plots of expression levels revealed that the seven genes related to ferroptosis, which were co-differentially expressed in MI and IS, exhibited significant differential expression between diseased and normal samples in both the MI-merged dataset (Fig. 3A) and the IS-merged dataset (Fig. 3B). Next, we conducted ROC validation on the combined MI and IS datasets to assess the diagnostic impact of the seven FRGs co-differentially expressed in MI and IS. These seven genes have a discernible diagnostic effect on both MI and normal samples (Fig. 3C) and a specific diagnostic effect on both IS and normal samples (Fig. 3D).

**Fig. 4:**
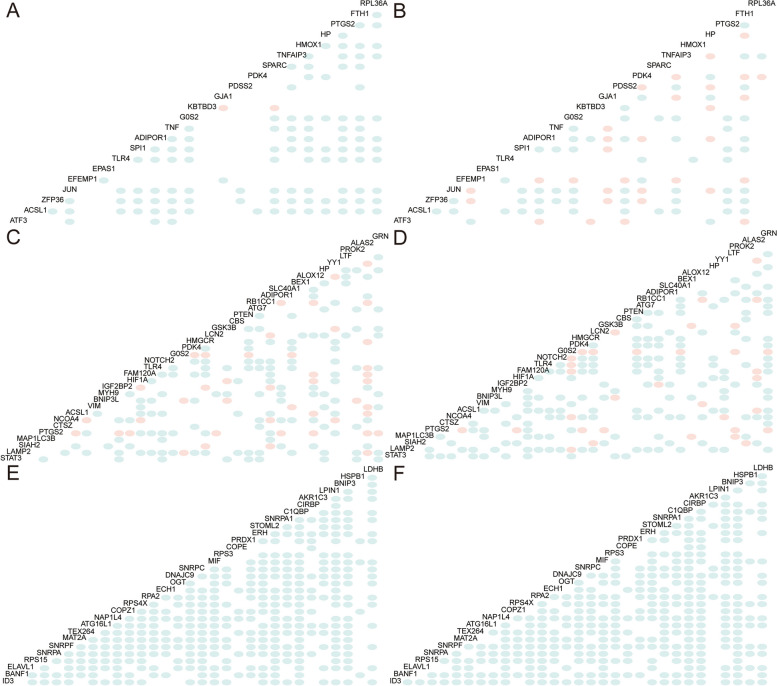
Correlation analysis of FRGs expressed differentially is conducted using the combined MI and IS dataset. **A** Correlation analysis was conducted on the gene expression levels of 22 differentially expressed FRGs in MI disease samples. Positive correlations are represented in blue and negative correlations are represented in pink. **B** In normal samples, a correlation analysis was conducted on the gene expression levels of 22 FRGs that were differentially expressed in MI. **C** Correlation analysis was conducted on the gene expression levels of 35 IS FRGs that exhibited increased expression in disease samples. **D** In normal samples, a correlation analysis was conducted on the expression levels of 35 differentially expressed IS genes that are related to ferroptosis. **E** In disease samples, a correlation analysis was conducted on the gene expression levels of 31 downregulated IS FRGs. **F** In normal samples, a correlation analysis was conducted on the gene expression levels of 31 downregulated IS FRGs. MI: Myocardial Infarction. IS: Ischemic Stroke. FRGs: Ferroptosis-related Genes

A total of 22 genes related to ferroptosis showed differential expression between MI and normal samples. We examined the correlation between the expression levels of these 22 genes in the normal and MI samples. The findings indicate that the majority of differentially expressed FRGs exhibited a positive correlation in MI samples (Fig. 4A), and a negative correlation in normal samples (Fig. 4B).

A total of 65 genes related to ferroptosis were differentially expressed between the IS and normal samples. Among these genes, 35 were upregulated and 31 were downregulated. Next, we examined the association between the expression levels of 35 distinct FRGs that showed increased expression, and 31 FRGs that showed decreased expression in both normal and MI samples. The findings indicated that the majority of FRGs that exhibited increased expression displayed favorable associations in both IS samples (Fig. 4C) and normal samples (Fig. 4D). FRGs expressed in a distinct manner, exhibiting reduced expression, exhibited positive correlations in nearly all of the IS samples (Fig. 4E) and in almost all of the normal samples (Fig. 4F).

**Fig. 5:**
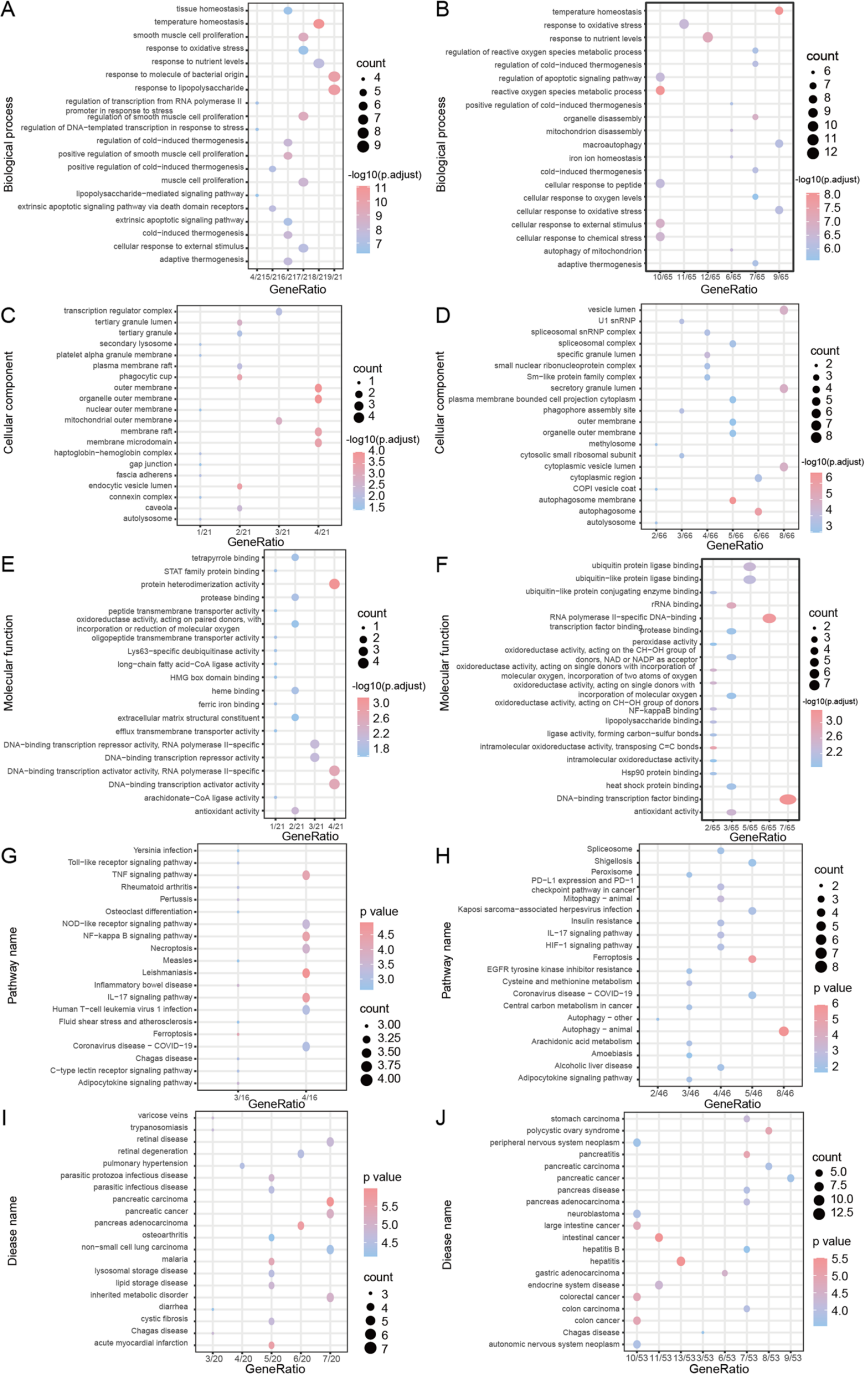
Characterizing biological features of genes showing differential expression using the combined MI and IS dataset. **A** The significance level is represented by the node color, while the number of genes included in the current GO Term is represented by the node size. The horizontal axis represents generation, and the vertical axis represents GO terms in BP enrichment analysis of MI DEGs. **B** Enrichment analysis of BP in GO terms for DEGs from IS. **C** Conducting CC enrichment analysis on the GO terms of MI DEGs. **D** Conducting CC enrichment analysis on the GO terms of IS genes that are expressed differentially. **E** Performing MF enrichment analysis on the GO terms of DEGs in MI. **F** Conducting MF enrichment analysis on the GO terms of DEGs in IS. **G** Enrichment analysis of MI's DEGs using KEGG. **H** KEGG enrichment analysis of the differentially expressed genes in IS. **I** Disease enrichment analysis of the differentially expressed genes in MI. **J** Disease enrichment analysis of the differentially expressed genes in individuals with IS. MI: Myocardial Infarction. IS: Ischemic Stroke. GO: Gene Ontology. KEGG: Kyoto Encyclopedia of Genes and Genomes. BP: Biological Process. CC: Cellular Component. MF: Molecular Function. DEGs: Differentially Expressed Genes

The GO functional annotation of MI DEGs in the merged datasets revealed that these genes were primarily enriched in various biological processes, including tissue and temperature homeostasis, and smooth muscle cell proliferation (Fig. 5A, Additional file 6: Table S5). Moreover, in terms of cellular components, they were associated with transcription regulator complexes, tertiary granule lumens, and tertiary granules (Fig. 5C). Furthermore, these genes exhibited molecular functions, such as tetrapyrrole binding, STAT family protein binding, and protein heterodimerization activity (Fig. 5E). KEGG Pathways were enriched in *Yersinia* infection, toll-like receptor signaling pathway, TNF signaling pathway, and other pathways (Fig. 5G, Additional file 7: Table S6). Finally, they were also associated with diseases such as varicose veins, trypanosomiasis, and retinal diseases (Fig. 5I).

Functional annotation of GO revealed that the DEGs in the combined dataset were primarily enriched in biological processes such as temperature regulation, oxidative stress response, and nutrient-level response (Fig. 5B, Additional file 8: Table S7). They were also associated with cellular components such as the vesicle lumen, U1 snRNP, and the spliceosomal snRNP complex (Fig. 5D). Additionally, these genes exhibited molecular functions such as ubiquitin protein ligase binding, ubiquitin-like protein ligase binding, and ubiquitin-like protein-conjugating enzyme binding (Fig. 5F). Furthermore, they were enriched in KEGG pathways such as Spliceosome, Shigellosis, and Peroxisome (Fig. 5H, Additional file 9: Table S8). Finally, these genes were linked to diseases, such as stomach carcinoma, polycystic ovary syndrome, and peripheral nervous system neoplasms (Fig. 5J).

## GSEA and GSVA enrichment

**Fig. 6:**
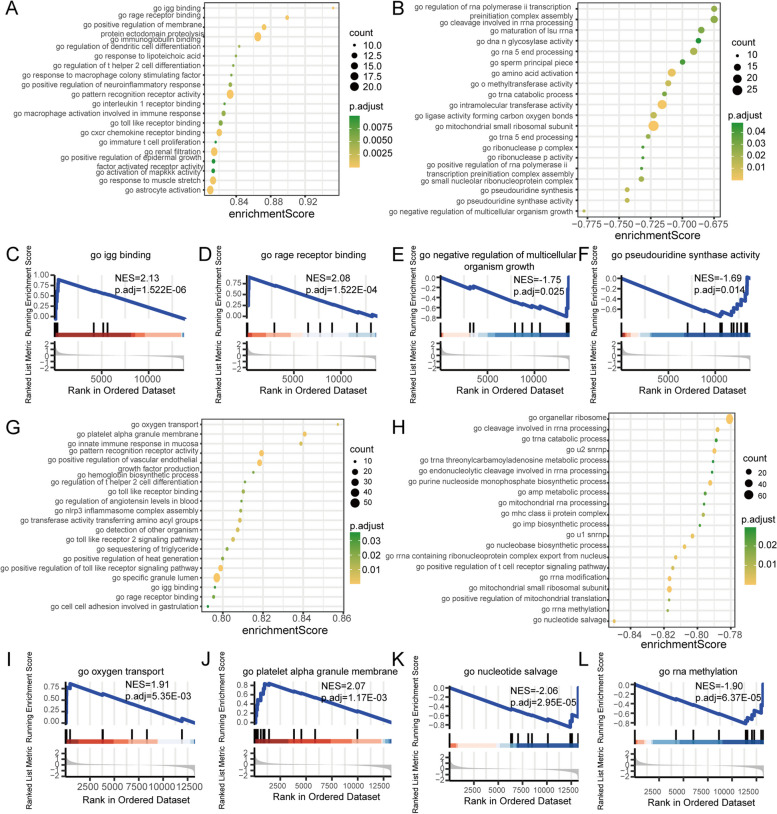
GSEA of the combined dataset of MI and IS. **A** GSEA reveals the activation of biological processes in patients with MI, with enrichment scores plotted on the horizontal axis, GO terms on the vertical axis, a color table indicating p-values, and node size indicating the number of enriched genes. **B** GSEA of biological processes suppressed in patients with myocardial infarction. **C** Igg binding demonstration in the enrichment results in A. **D** Rage receptor binding demonstrated in the enrichment results in A. **E** Negative regulation of multicellular organism growth demonstrated in enrichment results in B. **F** The enrichment results showed the presence of pseudouridine synthase activity in B. **G** GSEA of biological processes activated in patients with IS. **H** GSEA of biological processes inhibited in patients with IS. **I** Oxygen transport demonstrated in the enrichment results in G. **J** Platelet alpha granule membrane demonstrated in the enrichment results in G. **K** Nucleotide salvage display demonstrated in the enrichment results in H. **L** RNA methylation display in enrichment results in H. GSEA: Gene Set Enrichment Analysis. MI: Myocardial Infarction. IS: Ischemic Stroke

GSEA was performed on the merged MI and IS datasets to investigate the correlation between expressed and involved biological processes, affected cellular components, and molecular functions in disease and control samples. Promoted biological processes in MI disease samples (Fig. 6A, Additional file 10: Table S9) included igg binding (Fig. 6C), rage receptor binding (Fig. 6D), positive regulation of membrane protein ectodomain proteolysis, immunoglobulin binding, and regulation of dendritic cell differentiation. Conversely, inhibition of biological processes in MI disease samples (Fig. 6B) comprised negative regulation of multicellular organism growth (Fig. 6E), pseudouridine synthase activity (Fig. 6F), pseudouridine synthesis, small nucleolar ribonucleoprotein complex, and positive regulation of RNA polymerase ii transcription pre-initiation complex assembly. Promoted biological processes in the IS disease samples (Fig. 6G, Additional file 11: Table S10) included oxygen transport (Fig. 6I), platelet alpha granule membrane (Fig. 6J), innate immune response in the mucosa, pattern recognition receptor activity, and positive regulation of vascular endothelial growth factor production. Conversely, the inhibition of biological processes in IS disease samples (Fig. 6H) involved nucleotide salvage (Fig. 6K), RNA methylation (Fig. 6L), positive regulation of mitochondrial translation, mitochondrial small ribosomal subunit, and RNA modification, among others.

**Fig. 7:**
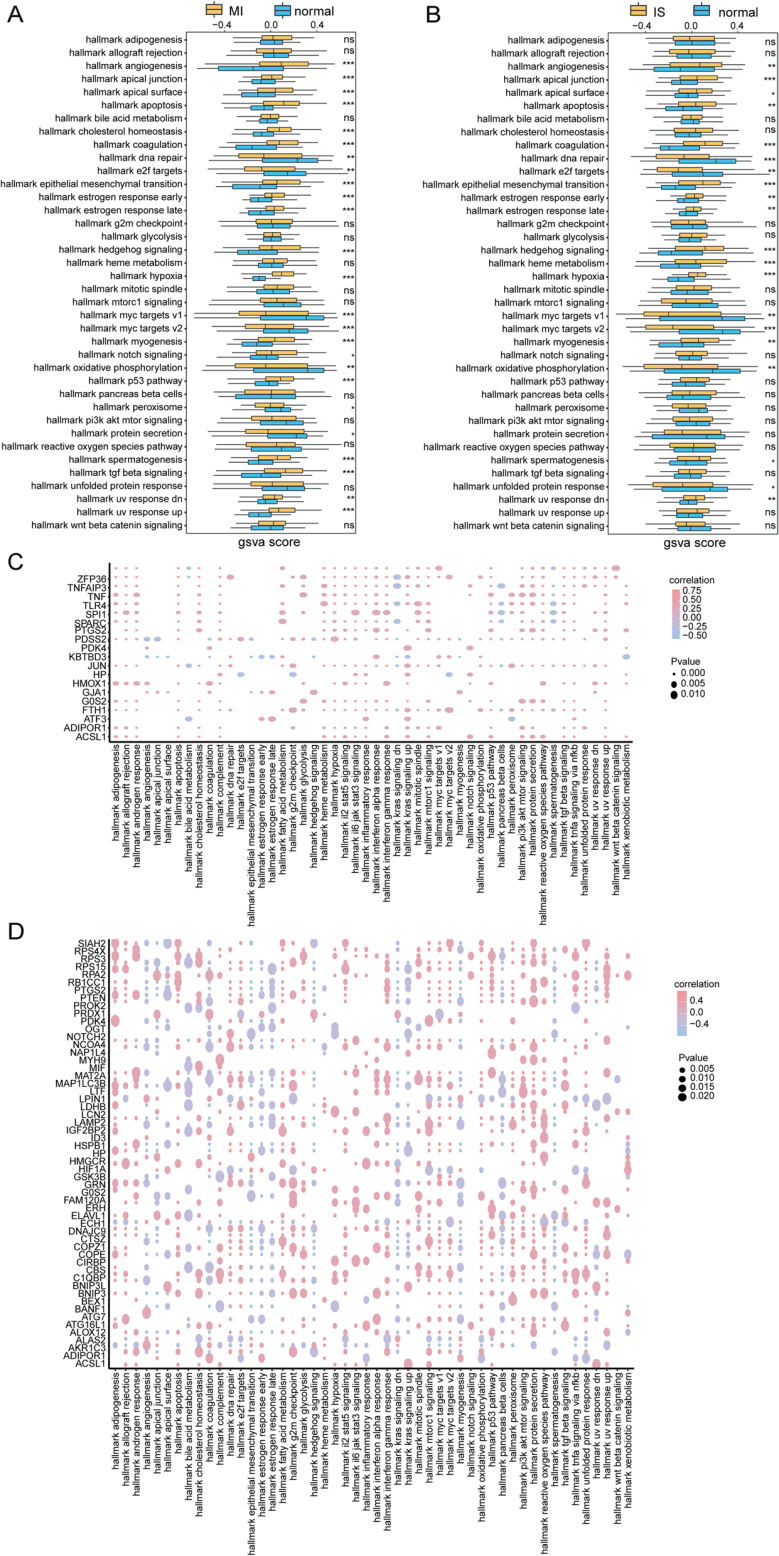
GSVA of the combined MI and IS datasets. **A** The hallmark distinction between MI and normal samples is represented by coordinates, wherein the hallmark is on the horizontal axis and the GSVA score is on the vertical axis. Cluster1 is indicated in pink, while cluster2 is indicated in blue. **B** The difference in hallmark between IS and normal samples. Significance levels are denoted as *p < 0.05; **p < 0.01; and ****p < 0.001. **C** Association of MI ferroptosis-related differential genes with characteristic on the horizontal axis and characterized genes on the vertical axis. Node size signifies the degree of significance, and node color signifies the degree of correlation. **D**. Correlation of IS ferroptosis-related differential genes with hallmark. Significance levels are denoted as *p < 0.05; **p < 0.01; and ****p < 0.001. GSVA: Gene Set Variation Analysis. MI: Myocardial Infarction. IS: Ischemic Stroke

To investigate the functional disparities between the disease and control samples in the merged MI and IS datasets, we conducted GSVA. The results revealed that MI samples exhibited significant activation of hallmark angiogenesis, hallmark apical junctions, hallmark apical surfaces, hallmark apoptosis, and other biological processes (Fig. 7A, Additional file 12: Table S11). Additionally, IS samples showed significant activation of hallmark hedgehog signaling, hallmark heme metabolism, and hallmark hypoxia (Fig. 7B, Additional file 13: Table S12). Furthermore, we computed the connections between differentially expressed genes in relation to ferroptosis and hallmark biological processes in 22 MI cases. The findings indicate that secreted protein acidic and rich in cysteine (SPARC) demonstrated a significantly positive correlation with hallmark glycolysis, hallmark 16 JAK/STAT3 signaling, hallmark inflammatory response, hallmark interferon alpha response, and hallmark interferon gamma response. Additionally, SPARC exhibited a significant negative correlation with hallmark pancreatic beta cells and spermatogenesis (Fig. 7C). Correlation analysis between 65 IS genes associated with ferroptosis and hallmark biological processes revealed that ACSL1 exhibited a significant positive correlation with upregulated hallmark KRAS signaling, downregulated hallmark uv response dn, and ADIPOR1. In contrast, the hallmark e2f targets, hallmark interferon-alpha response, hallmark oxidative phosphorylation, and hallmark unfolded protein response exhibited a significant negative correlation (Fig. 7D).

### Co-expression modules for MI and IS

**Fig. 8:**
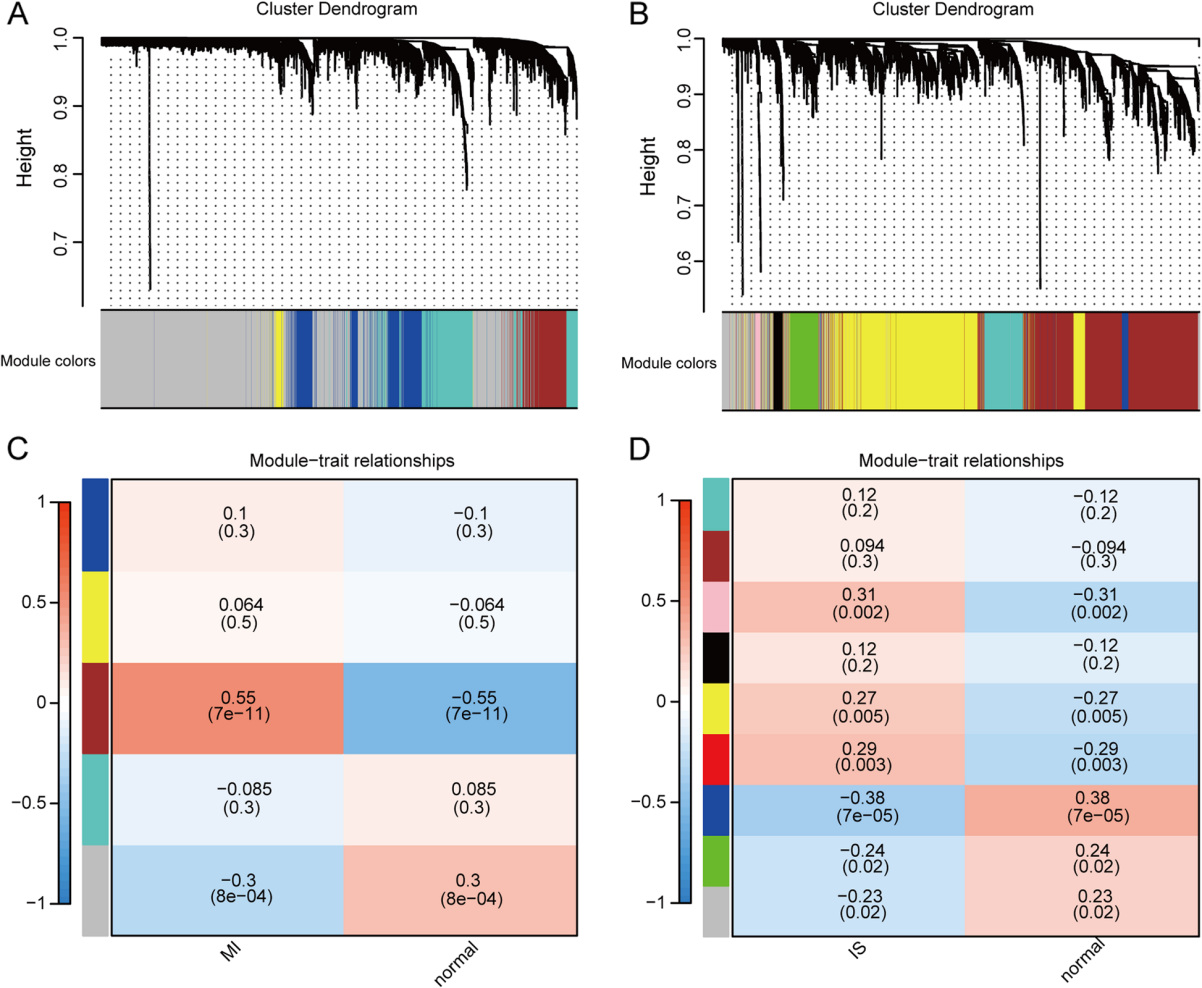
WGCNA of the combined MI, IS-based datasets. **A** Identification of MI-associated co-expression modules. **B** Identification of IS-associated co-expression modules. **C** The MI-associated module characterized genes are correlated with variables, represented by Pearson correlation coefficients and corresponding p values. Positive correlation is indicated in red, while negative correlation is indicated in blue. The clinical variables are plotted on the horizontal axis and the module characterized genes on the vertical axis. **D** Correlation between IS-associated module characterized genes and variables. WGCNA: Weighted Gene Co-expression Network Analysis. MI: Myocardial Infarction. IS: Ischemic Stroke

WGCNA was applied to the integrated MI datasets. To ensure a topology without scaling, the recommended soft threshold for scale-free R was 0.80 (Additional file 14: Figure S2A). The WGCNA identified five modules in the integrated MI dataset. Each gene module is indicated in a different color. To evaluate the correlation between each module and disease, heat maps were generated using Spearman’s correlation coefficients, illustrating the module-feature relationships (Fig. 8A). The modules named “gray” and “brown” strongly correlated with MI and were chosen as MI-associated modules (gray module, R = 0.3, *P* = 8e-04;’ brown module, R = 0.55, *P* = 7e-11). (Fig. 8C). Furthermore, we graphed the gene importance compared to the module affiliation for the “gray” (Additional file 14: Figure S2B) and “yellow” (Additional file 14: Figure S2C) modules individually. Scatter plot of gene significance vs. module membership. The total number of MI-associated genes was 3407.

WGCNA was used to identify gene modules linked to IS using integrated IS datasets. To ensure a scale-free topology (Additional file 14: Figure. S2D), the ideal soft threshold for scale-free R was determined to be 0.80. WGCNA identified nine modules in the integrated IS dataset, wherein each color corresponded to a distinct gene module. To evaluate the correlation between each module and disease, heat maps illustrating the module-feature relationships were generated using Spearman correlation coefficients (Fig. 8B). The IS-associated modules, namely the pink module (R = 0.31, *P* = 0.002) and blue module (R = 0.38, *P* = 7e-05), exhibited a strong correlation with IS (Fig. 8D). Furthermore, we graphed scatter plots displaying the correlation between gene significance and module membership for the “pink” (Additional file 14: Figure S2E) and "blue" (Additional file 14: Figure S2F) modules, respectively. We obtained a total of 3412 genes related to MI.

### Shared gene and network analysis of MI and IS

**Fig. 9:**
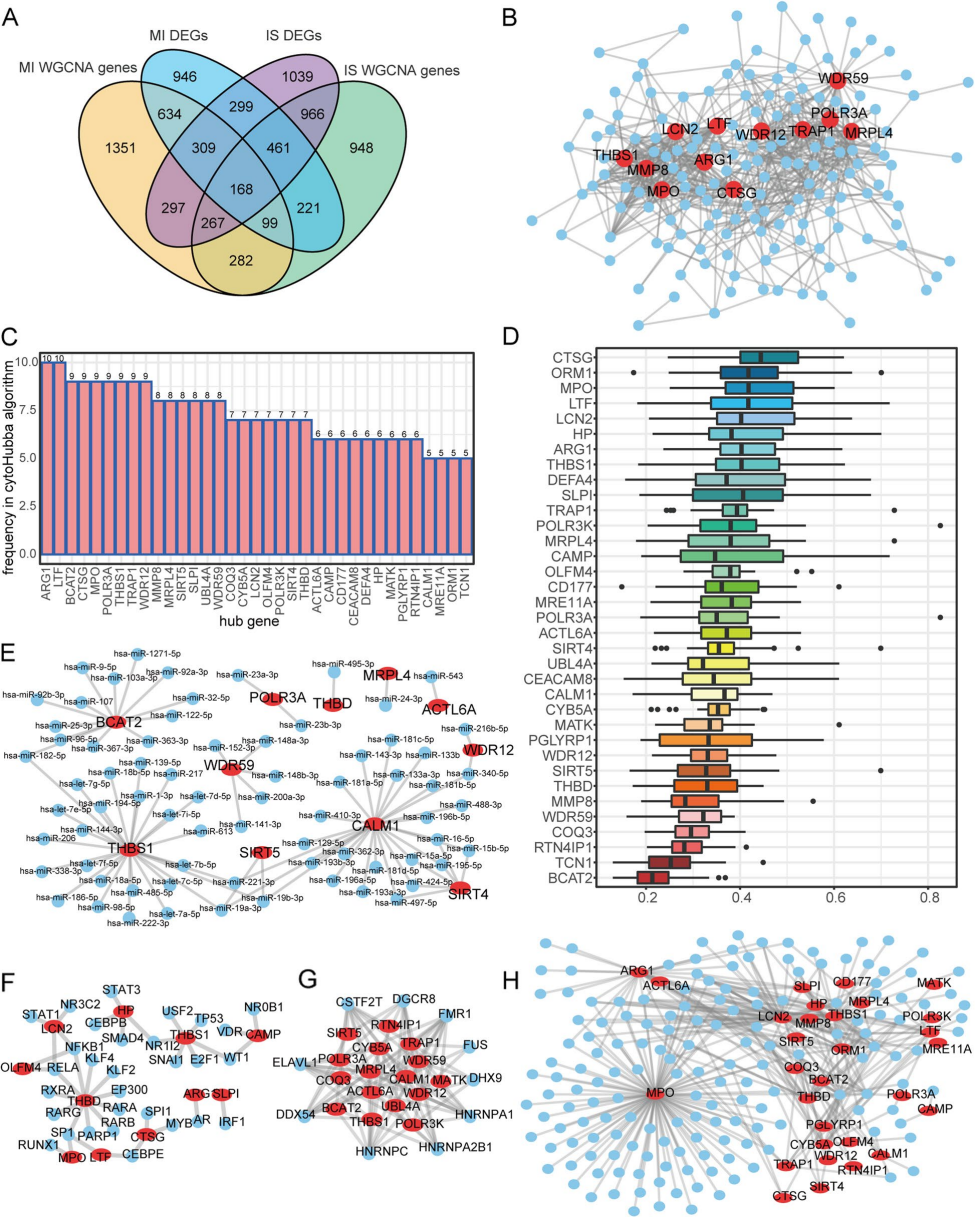
MI and IS shared genes and network analysis. **A** Wayne diagram displays the genes shared by MI and IS. Genes co-expressed with MI are shown in yellow, MI DEGs in blue, IS DEGs in purple, and IS co-expressed genes in green. **B** The PPI network of common genes. The shared genes are represented as blue nodes, whereas the shared genes with a greater number of shared genes in the medium degree are depicted in red. **C** Gene frequencies in the 12 algorithms are displayed in a table with genes and frequencies represented on the horizontal and vertical axes, respectively. **D** Gene similarity scores of hub genes in the PPI network of DEGs are represented by GO semantic similarity. The level of similarity is shown on the horizontal axis and genes are displayed on the vertical axis. **E** The network of hub genes and miRNAs is represented by mRNA-miRNA. Hub genes and miRNAs are depicted as red and blue nodes, respectively. **F** Network of hub genes and TFs is represented by mRNA. Hub genes and transcription factors are depicted as red and blue nodes, respectively. **G** The network of hub genes and RBPs consisting of red nodes representing hub genes and blue nodes representing RBPs. **H** The network of hub genes and drugs is represented by red and blue nodes for hub genes and drugs, respectively. MI: Myocardial Infarction. IS: Ischemic Stroke. PPI: protein–protein interaction. GO: Gene Ontology. RBPs: RNA-binding protein. DEGs: Differentially Expressed Genes

To identify shared genes between MI and IS, we extracted co-expressed genes associated with diseases in MI and IS from the combined datasets. Subsequently, we identified 3137 and 3806 genes that were differentially expressed in MI and IS, respectively. By intersecting the set of genes co-expressed with diseases and the set of genes differentially expressed in MI and IS, we identified 168 genes shared between MI and IS (Fig. 9A).

To examine the interactions among the DEGs, we built a PPI network using 168 common genes associated with MI and IS. The PPI network comprised 653 pairs of interactions and 163 shared genes. This network was visualized using Cytoscape. WDR59, ARG1, and MPO had the highest number of gene interactions (29, 29, and 28, respectively) (Fig. 9B).

Hub genes were extracted by calculating the top 30 nodes in each of the 12 algorithms in CytoHubba and selecting 35 genes that appeared in at least 5 algorithms (Fig. 9C). Using the R package “GOSemSim” [39], we calculated the semantic similarity of hub genes in Go. These findings indicated that CTSG, ORM1, and MPO exhibited functional correlations with multiple genes (Fig. 9D).

We predicted the miRNAs that interacted with 35 hub genes. Subsequently, we utilized Cytoscape to visually represent the network of mRNA-miRNA interactions (Fig. 9E). miRNAs are represented as sky-blue oval blocks in the network of mRNA-miRNA interactions, whereas mRNAs are depicted as red dots. The mRNA-miRNA interaction network reveals that our network comprised 11 hub genes and 71 miRNA molecules, forming a total of 84 mRNA-miRNA interaction pairs.

Using Cytoscape (Fig. 9F), we visualized the interaction relationship data of 11 hub genes and 30 TFs in the mRNA-TF network that we constructed. The mRNA-TF interaction network contained 39 pairs of mRNA-TF interactions.

A network of hub genes was built and 10 RNA-binding proteins (RBPs) corresponding to 16 hub genes were identified (Fig. 9G). The two RBPs, CSTF2T and FMR1, simultaneously target 11 crucial genes. The mRNA-RBP interaction network contained 81 pairs of mRNA-RBP interactions.

In the mRNA-drug interaction network (Fig. 9H), we built a network of hub genes associated with mRNA drugs and discovered 163 potential drugs or molecular compounds linked to 30 hub genes. We discovered that C006780 simultaneously targeted 20 hub genes. The network of interactions between the mRNA and drugs consisted of a grand total of 349 pairs.

### Construction of a diagnostic model related to Ferroptosis

**Fig. 10:**
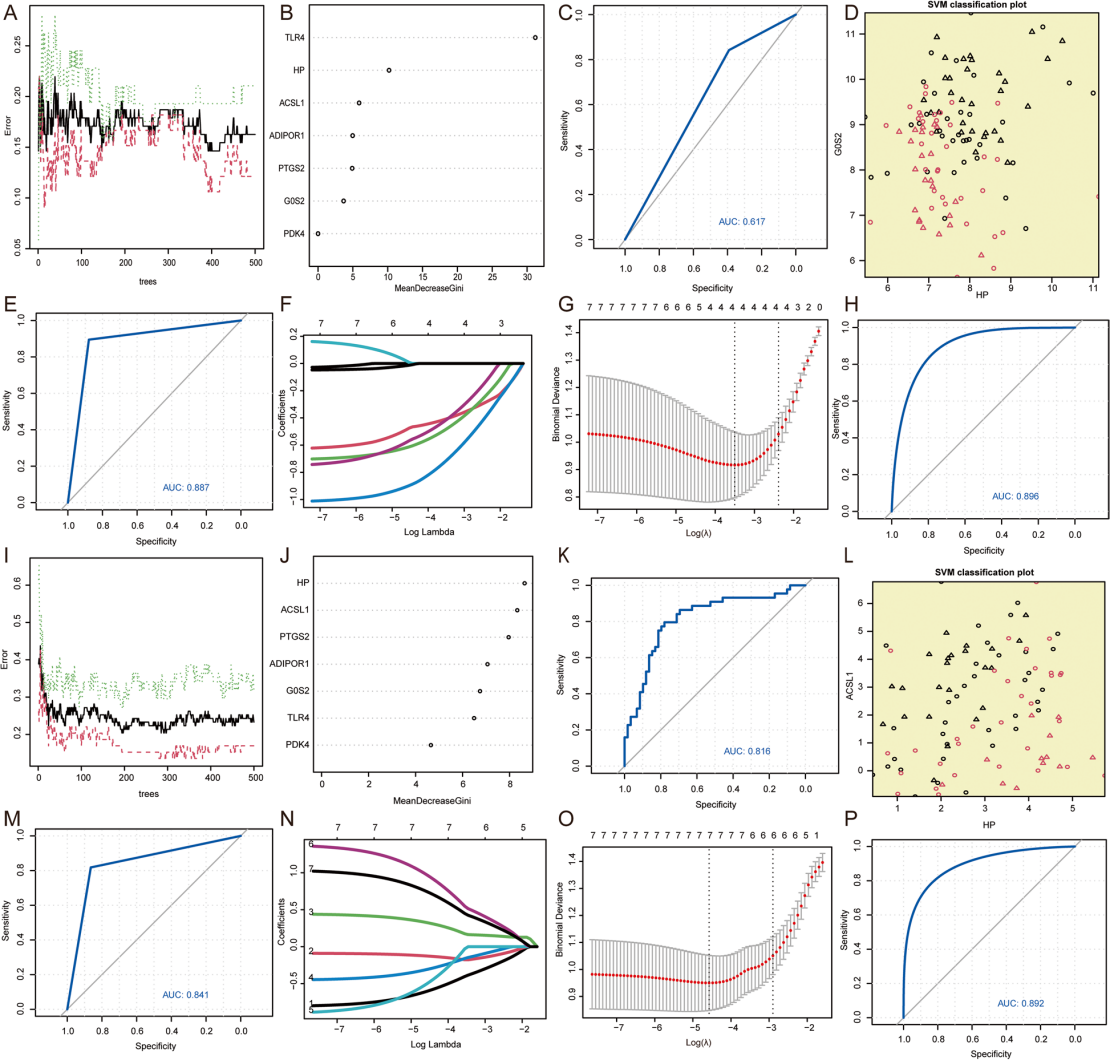
Feature selection screening for diagnostic markers. **A** Model training error plot for the MI RF algorithm. **B** RF model displays 7 genes related to ferroptosis in MI, arranged in descending order of MeanDecreaseGini, which are co-differentially expressed by MI and IS. **C** The ROC curves for diagnostics in the training set for MI used by RF model. **D** SVM Classification Surface Visualization in MI, with circles denoting the support vectors outside the data samples, and triangles indicate support vectors in the data. **E** The ROC curves for diagnostics in the training set for MI used by SVM. **F** The merged datasets were used to generate diagnostic model plots for LASSO regression of DEGs in MI. **G** LASSO variable trajectory plots of MI's diagnostic model for differential genes. **H** The ROC curves for diagnostics in the training set for MI used by LASSO regression. **I** RF Model training error plot for the algorithm in IS. **J** The RF model displays 7 genes related to ferroptosis (in descending order of MeanDecreaseGini) that show co-differential expression in MI and IS. **K** The ROC curves for diagnostics in the training set for IS used by RF model. **L** SVM Classification Surface Visualization in IS. **M** The ROC curves for diagnostics in the training set for IS used by SVM. **N** IS LASSO regression model for diagnostics in the training set. **O** Trajectory plots of LASSO variables for IS differential gene diagnostic models. **P** ROC curves for IS's LASSO regression model for diagnostics in the training set. MI: Myocardial Infarction. IS: Ischemic Stroke. ROC: Receiver operating characteristic curve. LASSO: Least absolute shrinkage and selection operator. SVM: Support Vector Machine. RF: Random Forest

The diagnostic value of the seven FRGs codifferentially expressed in MI and IS in the integrated MI datasets was assessed by analyzing their expression using RF (Fig. 10A). MeanDecreaseGini indicates the decrease in the Gini coefficient of a node. We filtered the results of specific analyses using MeanDecreaseGini > 0 as a criterion because nodes with reduced Gini coefficients were more valuable. From the results presented in (Fig. 10B), we identified six diagnostic markers of MI derived from seven genes associated with ferroptosis that were co-differentially expressed in both MI and IS. The markers were determined using the RF algorithm. In addition, ROC curves were generated for the combined MI datasets using the established RF model (Fig. 10C), which yielded an AUC value of 0.617. Subsequently, we employed a SVM to screen the selected genes and construct an MI diagnostic model. The radial kernel function was utilized, and the optimal number of feature genes obtained from the seven FRGs codifferentially expressed in MI and IS was five. Among these, two were randomly selected to represent the support vectors in the data (Fig. 10D). Furthermore, we predicted and plotted ROC curves based on the established model using the integrated MI datasets (Fig. 10E), resulting in an AUC value of 0.887. In conclusion, a diagnostic model for MI was developed using LASSO regression analysis (Fig. 10F). This model identified four MI diagnostic markers from a set of seven FRGs that were differentially expressed in both MI and IS. Furthermore, we visualized the results of the LASSO regression and generated trajectory plots for the LASSO variables (Fig. 10G). ROC curves were predicted for the integrated MI datasets using the established LASSO regression model (Fig. 10H) and achieved an AUC value of 0.896.

To assess the diagnostic significance of the seven MI and IS co-DEGs related to ferroptosis in the combined IS dataset, we examined the expression of these genes using the RF algorithm (Fig. 10I). MeanDecreaseGini indicates a decrease in the Gini coefficient of a node. The more Gini coefficients are reduced by the nodes, the more valuable they are. We filtered the specific analysis results using MeanDecreaseGini > 6 as the criterion. The findings indicated (Fig. 10J) that we identified six diagnostic indicators for IS from seven FRGs that were codifferentially expressed by MI and IS using the RF algorithm. Subsequently, we used the established RF model to predict and visualize the ROC curves on the integrated IS datasets (Fig. 10K), achieving an AUC value of 0.816. Next, we employed an SVM to screen the selected genes and construct the IS diagnostic model, utilizing the radial as the kernel function. Among the seven FRGs co-differentially expressed by MI and IS, we identified five optimal feature genes, two of which were randomly chosen to demonstrate the support vectors in the data (Fig. 10L). Furthermore, we predicted and plotted ROC curves based on the model constructed using the integrated IS dataset (Fig. 10M), which yielded an AUC value of 0. 841.In conclusion, a diagnostic model for IS was developed using the LASSO regression analysis (Fig. 10N). All seven FRGs, which were codifferentially expressed in MI and IS, served as diagnostic markers for MI. Furthermore, we visualized the results of the LASSO regression and generated trajectory plots for the LASSO variables (Fig. 10O). Additionally, we predicted and plotted ROC curves for the integrated IS datasets using the established LASSO regression model (Fig. 10P) and achieved an AUC value of 0.892.

After evaluating the AUC values of the diagnostic models created using RF, SVM, and LASSO, LASSO regression analysis was chosen as the ultimate method for constructing the diagnostic models for both MI and IS. The MI diagnostic model comprised 4 hub genes (TLR4, ADIPOR1, G0S2, and HP), whereas the IS diagnostic model consisted of 7 hub genes (ACSL1, TLR4, ADIPOR1, G0S2, PDK4, HP, and PTGS2). These models were selected as the final models.

**Fig. 11:**
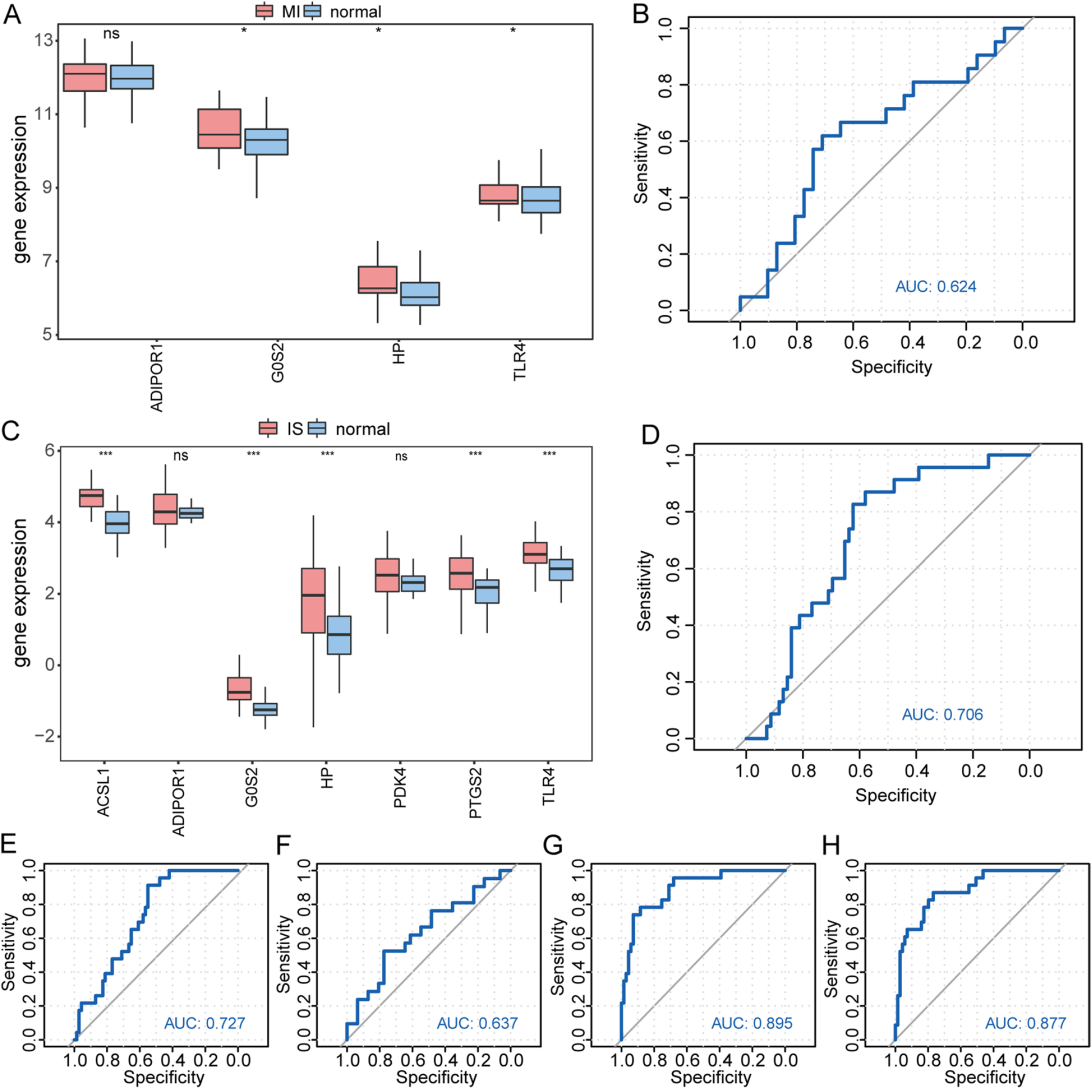
The validation set demonstrates the expression of important genes. **A** Plot comparing the MI hub genes categorized under MI grouping and Normal grouping in the GSE48060 dataset. **B** ROC curves for the LASSO regression model of MI in the diagnosis of the validation set GSE48060. **C** Plot comparing the IS hub genes grouped under IS subgroups and Normal subgroups in the GSE58294 dataset. **D** The ROC curves for the LASSO regression model of IS in the diagnosis of the validation set GSE58294. **E** ROC curves for HP in the GSE48060 validation set, showing the ROC curve for diagnosis in the same validation set GSE48060. **F** ADIPOR1 ROC curve of diagnosis in validation set GSE48060. **G** G0S2 ROC curve of diagnosis in validation set GSE58294. **H** ACSL1 ROC curve of diagnosis in validation set GSE58294. nsp > 0.05, *p < 0.05, **p < 0.01, and ***p < 0.001. MI: Myocardial Infarction. IS: Ischemic Stroke. ROC: Receiver Operating Characteristic. LASSO: Least Absolute Shrinkage and Selection Operator

To confirm whether these hub genes exhibited the same differential expression and expression patterns in other datasets, we generated box plots of the hub genes in the GSE48060 dataset by comparing the MI and normal samples (Fig. 11A). Of the four hub genes, three displayed significant differences in expression between the MI and normal samples, and their expression patterns aligned with the combined datasets. Additionally, we used the established LASSO regression model, which included the four hub genes, to predict the GSE48060 dataset and plotted ROC curves (Fig. 11B), resulting in an AUC value of 0.624. Furthermore, we independently verified the ROC curve of the hub genes in the model. The figure clearly illustrates that HP (Fig. 11E) and ADIPOR1 (Fig. 11F) exhibited a significant difference in expression between the MI and normal samples, demonstrating specific diagnostic implications.

We created box plots comparing the hub genes in the GSE58294 dataset between IS and normal samples (Fig. 11C). Of the seven hub genes, five exhibited notable variations in expression between IS and normal samples. Expression patterns were aligned with the combined datasets. Using the GSE58294 dataset, a LASSO regression model was constructed with seven hub genes, allowing expression prediction. Subsequently, an ROC curve (Fig. 11D) was generated with an AUC value of 0.706. Furthermore, we conducted ROC validation and observed that G0S2 (Fig. 11G) and ACSL1 (Fig. 11H) exhibited specific diagnostic impacts on both the IS and Normal samples.

### Immune infiltration analysis of combined datasets

**Fig. 12:**
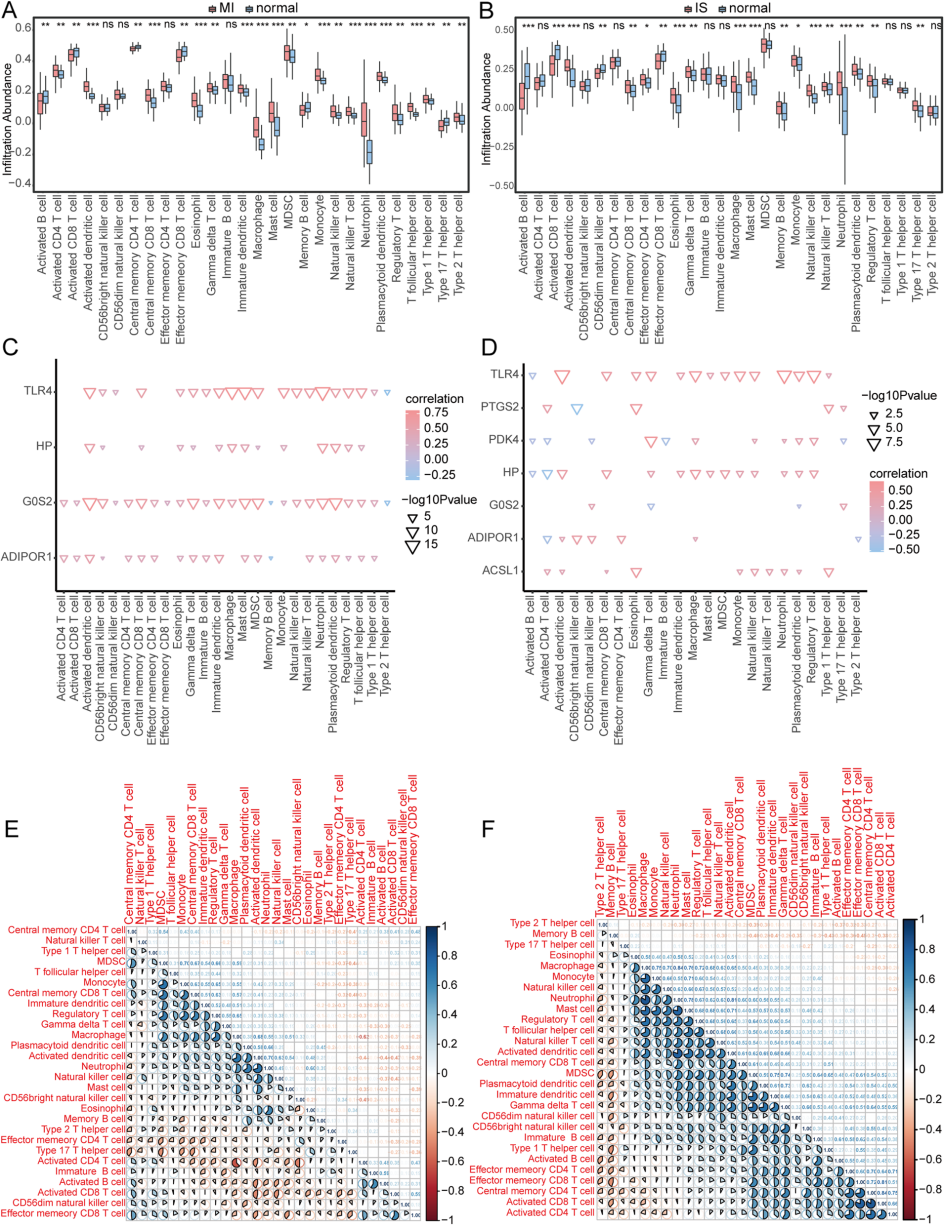
Comparison of immunological features in diseased and normal samples using ssGSEA. **A** The histogram displays the composition of immune cells in MI and normal samples. Cluster2 samples are represented by the color blue, while cluster1 samples are represented by the color pink. The immune cells are shown on the horizontal axis, and the cell content is represented on the vertical axis. **B** Comparison of immune cell composition between IS samples and normal samples using a histogram. **C** The relationship between identified genes and immune cells in MI samples is shown, with the size of the nodes representing the level of significance and the color indicating the correlation. Immune cells are represented on the horizontal axis, while characterized genes are represented on the vertical axis. **D** Relationship between identified genes and immune cells in IS samples. **E** The analysis of correlation between immune cell contents in MI samples shows that red represents negative correlation while blue represents positive correlation. **F** The correlation between the composition of immune cells in IS samples. MI: Myocardial Infarction. IS: Ischemic Stroke

The ssGSEA was used to evaluate the extent of immune cell infiltration in the combined datasets by comparing MI and IS samples to normal samples. The results indicate that the levels of immune cells, such as activated CD4 T cells, activated dendritic cells, central memory CD8 T cells, neutrophils, and T follicular helper cells, were higher than those in the normal samples. The levels of eosinophils, macrophages, natural killer cells, natural killer T cells, and regulatory T cells were notably elevated in MI samples (Fig. 12A). Compared to normal samples, IS samples exhibited significantly higher levels of these immune cells (Fig. 12B). The correlation between the expression levels of the identified genes and immune cells in MI and IS samples was calculated. The findings indicated a significant correlation between the expression of the identified genes, TLR4 and G0S2, and the presence of various immune cells in the MI samples (Fig. 12C). Similarly, the expression of the identified genes, TLR4 and HP, was significantly correlated with the content of multiple immune cells in IS samples (Fig. 12D). By separately calculating the correlation between the immune cell composition in MI and IS samples, we observed that monocytes, central memory CD8 + T cells, immature dendritic cells, regulatory T cells, gamma delta T cells in IS samples, and macrophages exhibited a positive correlation (Fig. 12E). In IS samples, there was a positive correlation between Type 2 T helper cells and macrophages, monocytes, natural killer cells, neutrophils, mast cells, regulatory T cells, T follicular helper cells, natural killer T cells, activated dendritic cells, central memory CD8 T cells, MDSC, plasmacytoid dendritic cells, immature dendritic cells, and gamma delta T cells. However, memory B cells were negatively correlated with most immune cells (Fig. 12F).

### Identification of patient subtypes by characterized genes

**Fig. 13:**
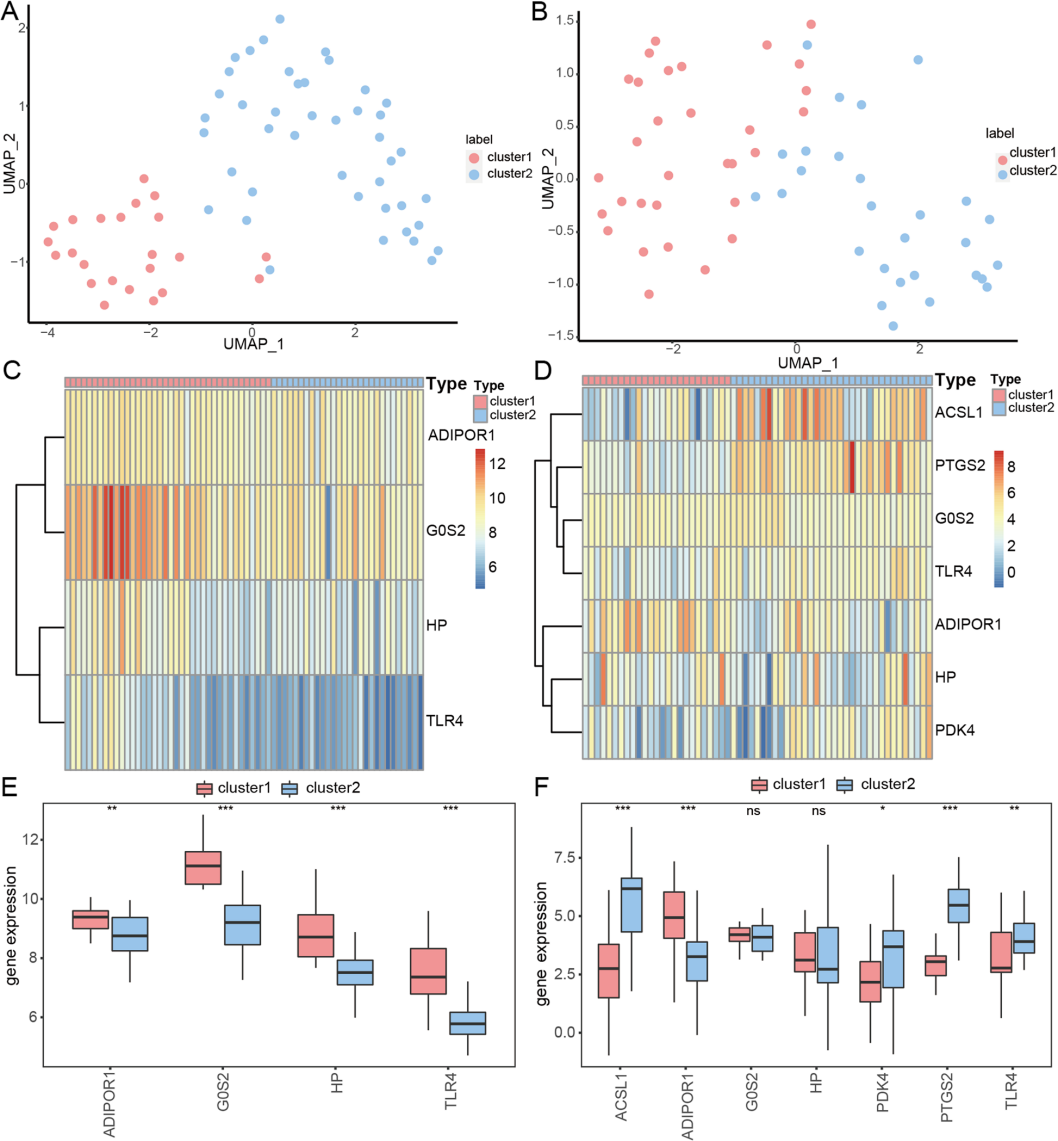
Characterization gene to patient clustering analysis. **A** UMAP clustering result plots of MI patients, cluster1 is indicated in pink and cluster2 is indicated in blue. **B** UMAP clustering result plots of IS patients. **C** A heatmap displaying the expression levels of MI characterized genes in two clusters, with cluster1 represented by pink and cluster2 represented by blue. **D** The expression levels of IS characterized genes in both clusters are represented by a heatmap, with cluster1 shown in pink and cluster2 shown in blue. **E** The expression levels of FRGs that are differentially expressed vary between cluster1 and cluster2 samples in MI patients. Cluster1 samples are indicated in pink, while cluster2 samples are indicated in blue. The hub gene is represented on the horizontal axis, while the gene expression level is shown on the vertical axis. **F** Expression levels of FRGs that are differentially expressed vary between samples from cluster1 and cluster2 in patients with IS. nsp > 0.05; *p < 0.05; **p < 0.01; ***p < 0.001. MI: Myocardial Infarction. IS: Ischemic Stroke. UMAP: Uniform Manifold Approximation and Projection. FRGs: Ferroptosis-related Genes

Using the UMAP method (Fig. 13A), two subtypes of patients, cluster1 and cluster2, were identified based on four hub genes. Cluster1 contained 23 samples whereas cluster2 contained 43 samples. Cluster analysis revealed variations in the identified genes between the two sample groups (Fig. 13C). This indicated that the four crucial genes exhibited significant differential expression in distinct subgroups of the MI combined dataset. The differences in the expression of the four hub genes between the two MI disease subtypes (cluster1 and cluster2) were examined, and the results are illustrated in a group comparison plot (Fig. 13E). Additionally, the featured genes exhibited differential expression between the two patient subtypes (*p* < 0.05).

Using the UMAP method (Fig. 13B), two subtypes of patients, namely cluster1 and cluster2, were identified based on the seven hub genes. There were 25 samples in Cluster1 and 34 samples in cluster2.The the clustering outcomes revealed variations in the identified genes between the two sample groups (Fig. 13D). This indicated that the seven hub genes exhibited differential expression in distinct subgroups of the combined IS dataset. The differences in expression of the seven hub genes between the two IS disease subtypes (cluster1 and cluster2) were examined, and the findings of the expression difference analysis are illustrated using a group comparison plot (Fig. 13F). Additionally, a significant proportion of the highlighted genes exhibited differential expression between the two patient subtypes (*p* < 0.05).

### Variations in immune traits among subcategories

**Fig. 14:**
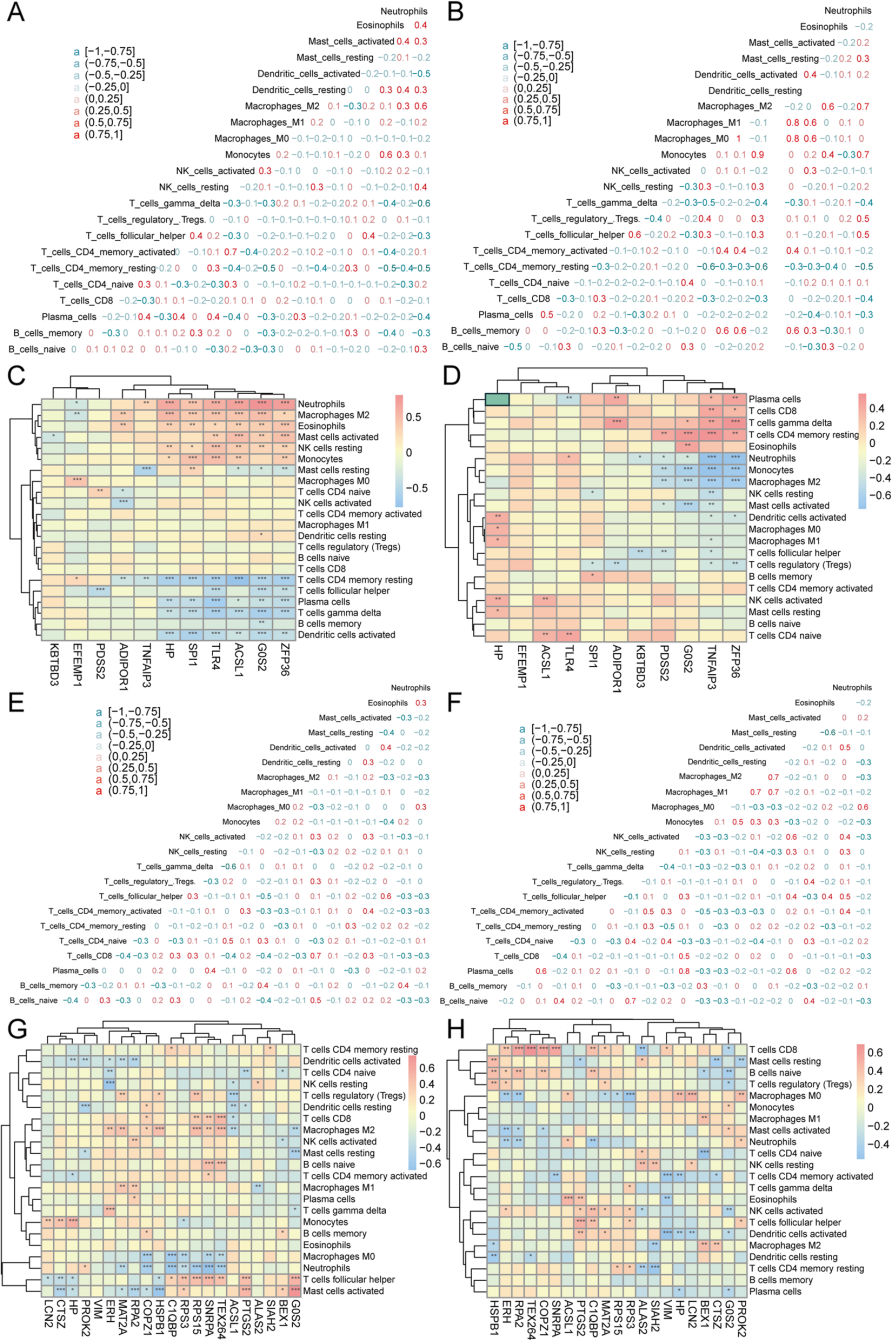
Immune characteristics between patients with different subtypes- CIBERSORTX. **A** The correlation between immune cell content in the cluster1 patient group with MI is indicated in pink for positive correlation and blue for negative correlation. **B** Correlation between immune cell composition in the cluster2 patient group and MI. **C** The correlation between immune cells and MI signature genes in the cluster1 patient group. The immune cells are shown on the vertical axis, while the signature genes are shown on the horizontal axis. Positive and negative correlations are indicated in red and blue, respectively. The size of the nodes represents the significance, and the color of the nodes represents the correlation. **D** Correlation between immune cells and MI signature genes observed in the cluster2 patient group. **E** The association between the number of immune cells in the cluster1 patient group and IS. **F** The relationship between the number of immune cells in the cluster2 patient group and IS. **G** The correlation between immune cells and genes characterized by IS in patients belonging to the cluster1 group. **H** The correlation between immune cells and genes characterized by IS in patients belonging to the cluster2 group. *p < 0.05; **p < 0.01; ***p < 0.001. MI: Myocardial Infarction. IS: Ischemic Stroke

To evaluate the extent of immune cell infiltration in the two distinct patient subtypes, the CIBERSORTx algorithm was employed. According to the findings, a notable distinction was observed in the correlation of immune cells between cluster1 and cluster2 patient groups (Fig. 14A and B) in individuals with MI. The correlation between the four hub genes and immune cell content in patients with the MI subtype was calculated separately. These findings indicate that the expression levels of multiple hub genes in patients in the cluster1 group were significantly positively correlated with NK cells at rest, macrophages M2, and neutrophils. Additionally, the expression levels of T CD4 memory cells at rest and in activated dendritic cells demonstrated a significant negative correlation with the expression levels of several hub genes (Fig. 14C). In the cluster2 patient group, expression of the central HP gene showed a significant positive correlation with the presence of various immune cells (Fig. 14D).

The correlation between immune cells in patients in cluster1 group (Fig. 14E) differed significantly from that in patients in cluster2 group (Fig. 14F). Associations between the seven hub genes and immune cell composition in patients with IS were also individually computed. These findings indicated a significant positive correlation between the expression level of the hub gene PDK4 and the presence of various immune cells in patients belonging to the CLUSTER1 group (Fig. 14G). In patients in cluster2 group, there was a strong positive correlation (Fig. 14H) between the abundance of various immune cells and the expression level of the central gene, TLR4.

### RT-qPCR validation of hub genes expression in cellular models and clinical disease blood specimens

**Fig. 15:**
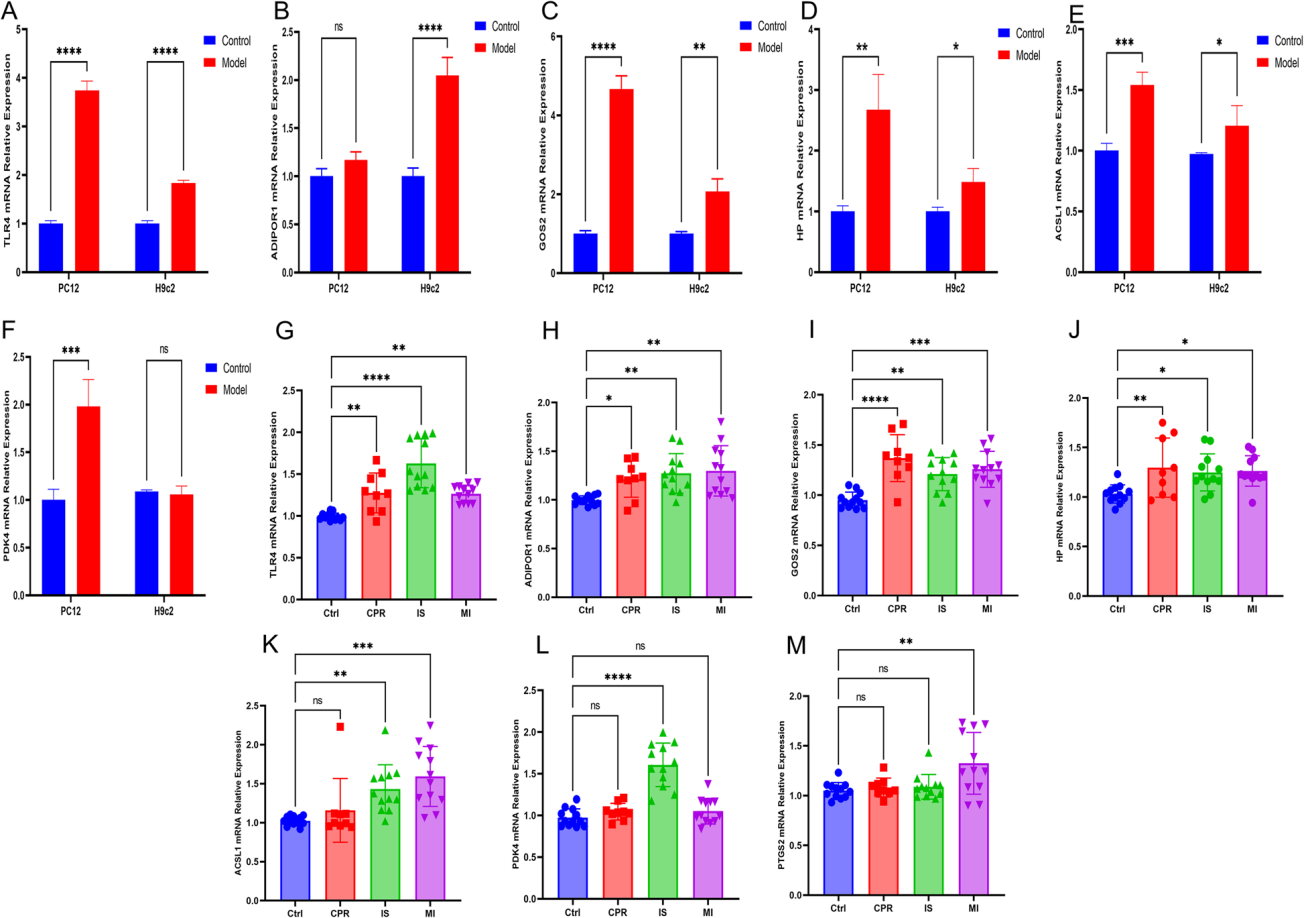
The expression of hub genes in cellular models and blood samples from clinical conditions. The mRNA expression levels of TLR4 **A**, **G**, ADIPOR1 **B**, **H**, GOS2 **C**, **I**, and HP **D**, **J**, ACSL1**E**, **K**, PDK4 **F**, **L**, and PTGS2 **M** were analyzed in PC12 cells, H9c2 cells, and blood samples obtained from patients undergoing CPR, acute IS, acute MI, as well as those undergoing normal physical examination. nsp > 0.05; *p < 0.05; **p < 0.01; ***p < 0.001; ****p < 0.0001. Ctrl: Control. CPR: cardiopulmonary resuscitation.IS: ischemic stroke. MI: myocardial ischemia

Compared to the controls, TLR4 mRNA expression was significantly increased in both cell models and blood specimens of clinical diseases (Fig. 15A and G). In the PC12 cell model, ADIPOR1 mRNA expression did not differ significantly from that in normal PC12 cells but was significantly higher in the H9c2 cell model and blood specimens from patients with clinical diseases (Fig. 15B and H). GOS2 mRNA expression was significantly higher in both cell models and blood specimens of patients with clinical diseases than in the control group (Fig. 15C and I). Similarly, HP mRNA showed a significant increase in both the cell model and blood specimens of patients with clinical diseases compared to the control group (Fig. 15D and J). ACSL1 mRNA expression was significantly elevated in the IS and MI groups compared to the control group, whereas there was no significant change in expression in the cardiopulmonary resuscitation group compared to the control group, and in the cellular model, where the expression of ACSL1 mRNA was significantly elevated (Fig. 15E and K), and the PDK4 mRNA expression was significantly higher in the IS group compared with the control group, whereas there was no significant change in expression in the MI and cardiopulmonary resuscitation(CPR) groups compared with the control group, and PDK4 mRNA expression was significantly higher in the PC12 cell model, whereas there was no significant change in expression in the H9c2 cell model (Figs. 15F and L). PTGS2 mRNA expression was significantly higher in MI group compared to controls, but no significant change in IS and CRR groups (Fig. 15M), In the cellular model, we confirmed by repeated verification of specimen RNA, primers and experimental conditions that PTGS2 mRNA expression was low in rats, and RT-qPCR was unable to detect the relevant expression of PTGS2 mRNA.

## Discussion

Effective preventative and curative techniques for cardio-cerebral damage resulting from cardiac arrest and complex cardio-cerebral vascular conditions are limited, leading to unsatisfactory treatment outcomes due to the limited timeframe and irreversible damage to heart muscle cells and neurons [40–42]. Despite functional and structural disparities, research indicates a remarkable resemblance in injury mechanisms between the heart and brain [1, 43]. However, the precise underlying mechanism remains unclear and requires further investigation. The involvement of ferroptosis in the progression of various diseases, including tumors and neurodegenerative diseases, has been previously reported [44]. An experiment conducted in mice with myocardial ischemia–reperfusion injury demonstrated an increase in non-heme iron content in the myocardium. Ferroptosis inhibitors alleviate ventricular remodeling and injury, indicating ferroptosis in the cardiomyocytes of mice with myocardial ischemia–reperfusion injury [45]. Following myocardial reperfusion, ACSL4 and iron levels increased, whereas GPX4 levels decreased [46]. Li et al. found that in reperfusion injury due to cardiac transplantation or cardiac coronary artery occlusion, cardiomyocytes undergo ferroptosis and trigger inflammation that exacerbates cardiac injury, and the use of an inhibitor of ferroptosis reduces cardiomyocyte ferroptosis [7]. Tuo et al. discovered that the effects of surgery on cerebral ischemia–reperfusion injury caused by middle cerebral artery occlusion (MCAO) were worsened by ferroptosis [47]. This study demonstrated that the suppression of ferroptosis reduced the damage caused by cerebral ischemia–reperfusion injury in the hippocampal neurons of mice in the MCAO model. This was achieved by enhancing GPX4 expression as described in a previous study [48]. These reports uncover the involvement of ferroptosis in cardiovascular disorders; however, additional investigations are required to fully understand its precise molecular mechanisms. This study aimed to establish a diagnostic model for the FRGs in patients with MI and IS. Multiple machine algorithms have been used to achieve this goal. Therefore, the best diagnostic model was selected for this study. In the future, bioinformatics methods will be used to analyze the biological processes of the hub genes, and further clinical specimens and cellular modeling experiments will be conducted to verify these findings. This study identified the hub genes related to ferroptosis in the process of cardiac ischemia–reperfusion injury and explored the biological mechanism of FRGs in this process.

In this study, we analyzed the expression of seven hub genes associated with ferroptosis in MI, IS, CRP, and physical examination populations, as well as in the H9c2 and PC12 cellular models, based on the aims of our study and the results of our validation dataset. We discovered that TLR4, ADIPOR1, G0S2, and HP play significant roles in the pathogenesis of MI and IS. The test set also confirmed that these hub genes have a diagnostic value for understanding the pathogenesis of MI and IS.

TLR4 (Toll-like receptor 4 (TLR4) plays a significant role in initiating the innate immune response and transmitting signals via a cascade of molecules [49]. The inflammatory response plays a crucial role following cardiac and cerebral ischemia/reperfusion injury [50, 51]. Recently, the association between TLR4 and inflammation was investigated. TLR4 exhibits the greatest expression among cardiac TLRs, Research has indicated that blocking the TLR4 signaling pathway can decrease the inflammatory response in heart muscle and potentially protect against further harm to the already injured myocardium [52]. Preclinical research has utilized genetically modified animal models to investigate the involvement of TLR4 in promoting inflammation and development of cardiac fibrosis and dysfunction [53]. Additionally, investigations have shown that during stroke, molecules typically contained within cells are released into the extracellular space owing to uncontrolled cell death. TLR4 has been identified as a potential target for stroke treatment because of its ability to bind circulating immune cells and activate an inflammatory response [54]. Additionally, the TLR4-NF-κB signaling pathway has been shown to alleviate inflammation in acute kidney injury [55]. TLR4 is a central gene involved in ferroptosis associated with ischemic stroke [56]. Recent findings also suggest that TLR4 plays a role in cardio-cerebral ischemic disorders, as evidenced by decreased expression of TLR4 in the cortex and hippocampus after cardiopulmonary resuscitation in rat models [57]. Taken together, these studies and our report further suggest an important role for TLR4 in cardiac ischemia–reperfusion injury; therefore, it is worthwhile to investigate the mechanisms of iron death and the role of TLR4 in this process.

ADIPOR1 (adiponectin receptor 1) plays a role in controlling fatty acid breakdown and glucose concentration. Interaction between ADIPOR1 and APPL1 diminishes the cardioprotective effects of adiponectin on myocardial ischemia/reperfusion injury in type 2 diabetic mice [58]. Autophagy-mediated ADIPOR1-AMPK signaling can suppress apoptosis in brain cells induced by cardiac arrest or cardiopulmonary resuscitation [59]. Additionally, the ADIPOR1-AMPK signaling pathway plays a crucial role in IS injury and can hinder the progression of cerebral injury after IS through lipocalin [60]. These findings align with our research and provide a basis for further investigation of shared pathways in cardiac and cerebral ischemic injuries.

G0S2 (G0/G1 switch 2) is primarily found in mitochondria and plays a role in promoting the extrinsic apoptotic signaling pathway. A retrospective clinical study indicated that low levels of G0S2 expression in peripheral blood may be a marker for MI [61]. Overexpression of G0S2 attenuates the decline in cardiomyocyte ATP and increases mitochondrial ATP production under hypoxia, whereas the knockdown of G0S2 expression leads to an increase in cardiomyocyte ATP decline [62]. No studies have investigated the relationship between G0S2 and IS have been published. However, in conjunction with the results of our study, we observed a significant increase in G0S2 expression in the peripheral blood of IS patients who underwent cardiopulmonary resuscitation. Further studies are required to elucidate the precise mechanisms.

ADIPOR1 and G0S2, as important findings in our study, provide a strong basis for us to study the common pathways of heart-brain ischemic injury in the direction of pre-glutamate metabolism and lipid metabolism. The aberrant expression of these two genes may play a key role in future clinical diagnosis and treatment, but further studies are still needed to verify their biological functions and potential clinical application value.

HP (haptoglobin) encodes a preprotein that binds to tetramers to produce binding beads. Bound adhesins can overutilize the available hemoglobin, thereby preventing oxidative damage caused by iron, inflammation, atherosclerosis, and cerebrovascular disease [63, 64]. The binding bead protein genotype is a consistent marker of coronary heart disease risk in individuals with elevated glycosylated hemoglobin levels [65]. In diabetic patients, the binding bead protein genotype is a major determinant of cardiovascular disease risk and can be used to predict CVD risk of cardiovascular disease in the presence of diabetes [66]. This suggests that HP is closely linked to the development of cardiovascular diseases, particularly ischemic diseases. Additionally, different HP genotypes are closely associated with atherosclerosis in patients with ischemic stroke, and the HP2-2 genotype serves as a genetic biomarker for precision medicine and personalized healthcare in stroke patients [67]. The MCAO model also showed that proteins attached to the binding beads had an effect on macrophage/microglia-induced inflammation and were able to protect the brain from ischemic injury. This attachment improves survival and motor function and reduces brain damage in rats. Therefore, HP may be an effective treatment for cerebral ischemia [68]. Along with the corresponding analysis of biological functions, this provides a direction for future investigations into the shared pathways of ischemic injury.

Using GO functional analysis, we examined the DEGs in the combined datasets and discovered that these genes shared numerous common functions in MI and IS, including oxidative stress and apoptosis. KEGG pathway enrichment analysis indicated that the DEGs were primarily enriched in MI and IS within pathways such as the Toll-like signaling pathway, IL-17 signaling pathway, Ferroptosis, and Adipocytokine signaling pathway. Various studies have demonstrated that resveratrol effectively improves acute MI through mechanisms associated with reduced oxidative stress and inflammation, potentially by affecting TLR4 expression [69]. Additionally, the IL-17 signaling pathway can influence the connection between multiple sclerosis and acute MI, making it a potential target for pharmacological intervention in acute MI [70]. Similarly, in acute IS, the upregulated expression of lncRNA ENSG00000226482 has been identified as a diagnostic and therapeutic biomarker. This effect is mainly achieved through activation of the adipocytokine signaling pathway [71]. The findings of these studies align with ours and provide direction for further investigation of the functions of upstream and downstream hub genes in ischemic injury.

We used ssGSEA to evaluate the levels of immune infiltration in the MI and IS datasets. Additionally, we investigated the features of distinct ferroptosis-related hub genes in various subtypes of MI and IS along with their immune profiles. The analysis revealed significant differential expression of most hub genes among the subtypes and substantial variation in immune cell correlations across different disease subtypes. During MI and IS, the infiltration of various immune cell types is strongly associated with the progression of cardio-cerebral vascular diseases [72–75]. Therefore, it is imperative to further investigate associated immune infiltration.

In the field of computational biology, the continuous progress of interactions prediction research has provided us with insights into the molecular mechanisms of MI and IS as well as potential biomarkers. In particular, mRNA-TF, miRNA-ncRNA, and mRNA-miRNA interaction prediction has become an area of great interest, which is crucial for revealing the interactions of miRNAs, non-coding RNAs, etc. with MI and IS. In future studies: we plan to further improve and develop more accurate interactions prediction models to better identify the effects of mRNA-miRNA-ncRNA interactions on MI and IS. In the future, we will explore more multi-omics data, including transcriptomics, proteomics, metabolomics data and single-cell multi-omics data [76], to further comprehensively understand the pathophysiological processes of MI and IS by using autocoders, non-negative matrix decomposition, conditional random field graph convolutional networks, network distance analysis and asymmetric autocoders for the framework of single-cell data analysis [77–80], and eventually We will provide better diagnostic and therapeutic methods for cardiac and cerebral ischemia injuries through functional validation and clinical practice.

In a previous study, we examined the biological mechanisms of the genes associated with glutamate receptors in MI and IS. Additionally, we identified glutamate receptor-related hub genes using LASSO regression analysis. However, we did not comprehensively analyze the biological processes involved in MI and IS [4]. Ferroptosis is a specific glutamate excitotoxic process caused by ROS aggregation [81]. This study utilized more relevant datasets and multiple machine algorithms(RF, SVM, LASSO, CIBERSORX, ESTIMATE analysis, and nonlinear dimensionality reduction algorithm, etc.) to further explore the role of ferroptosis in cardio-cerebral ischemia and the related biological processes during cardio-cerebral ischemia, and screened out ferroptosis -related hub genes by using the test datasets as well as the validation of the clinical specimens and the cellular models, which provided an effective guideline to further explore the common pathway of cardio-cerebral injury, and provided theoretical support for the subsequent exploration of common pathways of cardio-cerebral ischemia injury based on the processes of glutamate signaling and ferroptosis. Nevertheless, the current investigation has some shortcomings, and our confirmation of the hub genes was limited to the initial sample and cellular verification, necessitating further exploration of the precise mechanism in the forthcoming research. Owing to the limited data available in the current datasets, it was not possible to integrate the hub genes with clinical information to analyze their correlation with onset time, prognosis, and other factors. Therefore, to thoroughly investigate the mechanism of ischemic injury, we collected pertinent blood samples and clinical data, and prepared them for additional sequencing.

## Conclusions

Four ferroptosis-related hub genes, TLR4, ADIPOR1, G0S2, and HP, can be used as diagnostic markers of cardio-cerebral ischemic diseases and provide an effective guideline for further exploration of the common injury pathways of cardio-cerebral injuries.

### Supplementary Information

**Additional file 1:** **Table S1.** GEO Dataset Information list.

**Additional file 2:** **Table S2.** Ferroptosis-related genes.

**Additional file 3:** **Table S3.** Primer sequences for cell experiments.

**Additional file 4:** **Table S4.** Primer sequences for blood specimens.

**Additional file 5:** **Fig. S1.** Data processing for merged data sets. (A) Merging of the MI datasets GSE60993 and GSE66360. (B) Removing batch effects between MI datasets. (C)A significant batch effect between the MI combined data. (D) Gene expression profiling data with consistent expression levels after treatment. (E) Merging of the IS datasets GSE22255和GSE16561. (F) Removing batch effects between IS datasets. (G)A significant batch effect between the IS combined data. (H) Gene expression profiling data with consistent expression levels after treatment. MI: Myocardial Infarction. IS: Ischemic Stroke.

**Additional file 6:** **Table S5.** GO enrichment results of MI.

**Additional file 7:** **Table S6.** KEGG enrichment results of MI.

**Additional file 8:** **Table S7.** GO enrichment results of IS.

**Additional file 9:** **Table S8.** KEGG enrichment results of IS.

**Additional file 10:** **Table S9.** GSEA-MI.

**Additional file 11:** **Table S10.** GSEA-IS.

**Additional file 12:** **Table S11.** GSVA-MI.

**Additional file 13:** **Table S12.** GSVA-IS.

**Additional file 14:** **Fig. S2.** Construction of co-expression modules for MI and IS. (A) The optimal soft threshold for MI gene module scale-free R is 0.80 to ensure a non-scaling topology. (B) Plotting the scatter plot of gray module gene significance vs. module membership for MI. (C) Plotting the scatter plot of yellow module gene significance vs. module membership for MI. (D)The optimal soft threshold for IS gene module scale-free R is 0.80 to ensure a non-scaling topology. (E) Plotting the scatter plot of pink module gene significance vs. module membership for IS. (F) Plotting the scatter plot of blue module gene significance vs. module membership for IS. MI: Myocardial Infarction. IS: Ischemic Stroke.

## Data Availability

This study contains all data and materials that can be verified and accessed, as they have been deposited in online repositories. Detailed information about these repositories is available in the article or Additional Materia.

## Abbreviations

ROC: Receiver operating characteristic
MI: Myocardial ischemia
IS: Ischemic stroke
PPIs: Protein-protein interactions
TFs: Transcription factors
RBPs: RNA-binding proteins
ssGSEA: Single sample gene set enrichment analysis
UMAP: Uniform Manifold Approximation and Projection
FRGs: Ferroptosis-related genes
GEO: Gene Expression Omnibus
DEGs: Differentially expressed genes
PCA: Principal Component Analysis
GO: Gene Ontology
BP: Biological process
MF: Molecular function
CC: Cellular component
TF: Transcription Factor
KEGG: Kyoto Encyclopedia of Genes and Genomes
DO: Disease Ontology
GSEA: Gene Set Enrichment Analysis
GSVA: Gene Set Variation Analysis
LASSO: Least absolute shrinkage and selection operator
RF: Random Forest
SVM: Support Vector Machine
WGCNA: Weighted Gene Co-expression Network Analysis
AUC: Area under the curve
STRING: Search Tool for the Retrieval of Interacting Genes/Proteins
ENCORI: Encyclopedia of RNA Interactomes
CTD: Comparative Toxicogenomics Database
RT-qPCR: Real-time fluorescence quantitative PCR
TLR4: Toll-like receptor 4
ADIPOR1: Adiponectin receptor 1
GOS2: G0/G1 switch 2
HP: Haptoglobin
