# Supplementary material for: Integrative analyses and validation of ferroptosis-related genes and mechanisms associated with cerebrovascular and cardiovascular ischemic diseases
Source: BMC Genomics. 2023 Dec 4;24:731. doi: 10.1186/s12864-023-09829-w (PMC10694919; doi:10.1186/s12864-023-09829-w)
Supplement: Supplementary file 1 — Additional file 1: Table S1. GEO Dataset Information list. [file 12864_2023_9829_MOESM1_ESM.docx]

Table S1: GEO Dataset Information list.

| Number | GSE ID | GPL  Platform | Sequencing  Type | Molecular Type | Species Source | Tissue Source | Group | Sample Grouping and Size | Reference |
| --- | --- | --- | --- | --- | --- | --- | --- | --- | --- |
| 1 | GSE60993 | GPL6884 Illumina HumanWG-6 v3.0 expression beadchip | microarray | mRNA | Homo sapiens | Human blood | 4 | Samples：33； ST-elevation myocardial infarction  (STEMI, n=7)；  Non-ST-elevation MI (NSTEMI, n=10)；  unstable angina  (UA, n=9)；  normal control (n=7) | Expression pattern and diagnostic value of ferroptosis-related genes in acute myocardial infarction |
| 2 | GSE66360 | GPL570 [HG-U133_Plus_2] Affymetrix Human Genome U133 Plus 2.0 Array | microarray | mRNA | Homo sapiens | Circulating Endothelial Cells | 2 | Samples：99； acute myocardial infarction (n=49) ；  healthy control (n=50) | Identification and validation of senescence-related genes in circulating endothelial cells of patients with acute myocardial infarction |
| 3 | GSE48060 | GPL570 [HG-U133_Plus_2] Affymetrix Human Genome U133 Plus 2.0 Array | microarray | mRNA | Homo sapiens | Human blood | 2 | Samples：52； myocardial infaction patient groups (n=31) ；  healthy control  (n=21) | Predicting Diagnostic Gene Biomarkers Associated With Immune Infiltration in Patients With Acute Myocardial Infarction |
| 4 | GSE22255 | GPL570 [HG-U133_Plus_2] Affymetrix Human Genome U133 Plus 2.0 Array | microarray | mRNA | Homo sapiens | Peripheral blood | 2 | Samples：40；  IS patients  (n = 20)；  healthy control  (n =20) | Bioinformatics Identification of Ferroptosis-Related Biomarkers and Therapeutic Compounds in Ischemic Stroke |
| 5 | GSE16561 | GPL6883 Illumina HumanRef-8 v3.0 expression beadchip | microarray | miRNA | Homo sapiens | Human blood | 2 | Samples：63； IS patients  (n = 24) ；  healthy control  (n =39) | Bioinformatics Identification of Ferroptosis-Related Biomarkers and Therapeutic Compounds in Ischemic Stroke |
| 6 | GSE58294 | GPL570 [HG-U133_Plus_2] Affymetrix Human Genome U133 Plus 2.0 Array | microarray | mRNA | Homo sapiens | Human blood | 2 | Samples：92； cardioembolic stroke  (n = 69) ；  healthy control  (n =23) | Bioinformatics analysis and in vivo validation of ferroptosis-related genes in ischemic stroke |

IS: ischemic stroke.
