# Supplementary material for: Integrative analyses and validation of ferroptosis-related genes and mechanisms associated with cerebrovascular and cardiovascular ischemic diseases
Source: BMC Genomics. 2023 Dec 4;24:731. doi: 10.1186/s12864-023-09829-w (PMC10694919; doi:10.1186/s12864-023-09829-w)
Supplement: Supplementary file 3 — Additional file 3: Table S3. Primer sequences for cell experiments. [file 12864_2023_9829_MOESM3_ESM.docx]

Table S3. Primer sequences for cell experiments.

| Primer | Primer sequences (5’-3’) | Product length(bp) | | Annealing temperature (℃) |
| --- | --- | --- | --- | --- |
| β-actin F | GCCATGTACGTAGCCATCCA | 375 | 58.0 | |
| β-actin R | GAACCGCTCATTGCCGATAG |  |  |  |
| TLR4 F | CCAGAGCCGTTGGTGTATCT | 137 | 59.0 | |
| TLR4 R | GGCGATACAATTCGACCTGC |  |  |  |
| ADIPOR1F | TTGGAGGGTCATCCCATACG | 194 | 58.1 | |
| ADIPOR1R | TGGTCAAGATTCCCAGAAAGG |  |  |  |
| G0S2 F | TAAGGAGATGATGACGCAGAAGT | 98 | 60.0 | |
| G0S2 R | CCACTAGACCGAGCACCACA |  |  |  |
| HP F | AAGTGTGAGGCAGTGTGTGG | 215 | 60.3 | |
| HP R | TTGGCTGTCGCATTCTCACT |  |  |  |
| ACSL1 F | GAAGAACTGTGCAGGAACAAGGAT | 81 | 60.1 | |
| ACSL1 R | CTTCAAACCAGCATTCTTCCCAAG |  |  |  |
| PDK4 F | GAGTTCCACGAAAAGAGCCCA | 335 | 60.2 | |
| PDK4 R | TTTGCACACTCAAAGGCATCTTC |  |  |  |
| PTGS2 F | GTGGGATGACGAGCGACTGT | 171 | 60.1 | |
| PTGS2 R | GAGGCAATGCGGTTCTGATACT |  |  |  |
