# Supplementary material for: Integrative analyses and validation of ferroptosis-related genes and mechanisms associated with cerebrovascular and cardiovascular ischemic diseases
Source: BMC Genomics. 2023 Dec 4;24:731. doi: 10.1186/s12864-023-09829-w (PMC10694919; doi:10.1186/s12864-023-09829-w)
Supplement: Supplementary file 4 — Additional file 4: Table S4. Primer sequences for blood specimens. [file 12864_2023_9829_MOESM4_ESM.docx]

Table S4. Primer sequences for blood specimens.

| Primer | Primer sequences (5’-3’) | Product length(bp) | Annealing temperature (℃) |
| --- | --- | --- | --- |
| β-actin F | TGGCACCCAGCACAATGAA | 186 | 60.1 |
| β-actin R | CTAAGTCATAGTCCGCCTAGAAGCA |  |  |
| TLR4 F | CGCTTTCACTTCCTCTCACC | 125 | 59.8 |
| TLR4 R | GCTCGCTATCACCGTCTGA |  |  |
| ADIPOR1 F | CTTCTACTGCTCCCCACAGC | 196 | 58.6 |
| ADIPOR1 R | GACAAAGCCCTCAGCGATAG |  |  |
| G0S2 F | AGGAGATGATGGCCCAGAAG | 205 | 59.8 |
| G0S2 R | AGGGCTTGCTTCTGGAGAG |  |  |
| HP F | TGGCTATGTGGAGCACTCGG | 103 | 60.1 |
| HP R | TATCCACTGCTTCTCATTG |  |  |
| ACSL1 F | CCATGAGCTGTTCCGGTATTT | 93 | 60.0 |
| ACSL1 R | CCGAAGCCCATAAGCGTGTT |  |  |
| PDK4 F | CAATGGCACAAGGAATCATAG | 123 | 57 |
| PDK4 R | TCATCAGCATCCGAGTAGAAAT |  |  |
| PTGS2 F | TTCCTCCTGTGCCTGATGATT | 173 | 59.7 |
| PTGS2 R | AAACTGATGCGTGAAGTGCTG |  |  |
