# Supplementary material for: Integrative analyses and validation of ferroptosis-related genes and mechanisms associated with cerebrovascular and cardiovascular ischemic diseases
Source: BMC Genomics. 2023 Dec 4;24:731. doi: 10.1186/s12864-023-09829-w (PMC10694919; doi:10.1186/s12864-023-09829-w)
Supplement: Supplementary file 5 — Additional file 5: Fig. S1. Data processing for merged data sets. (A) Merging of the MI datasets GSE60993 and GSE66360. (B) Removing batch effects between MI datasets. (C)A significant batch effect between the MI combined data. (D) Gene expression profiling data with consistent expression levels after treatment. (E) Merging of the IS datasets GSE22255和GSE16561. (F) Removing batch effects between IS datasets. (G)A significant batch effect between the IS combined data. (H) Gene expression profiling data with consistent expression levels after treatment. MI: Myocardial Infarction. IS: Ischemic Stroke. [file 12864_2023_9829_MOESM5_ESM.zip › Additional file 5 Figure S1.docx]

**Fig S1. Data processing for merged data sets.** (A) Merging of the MI datasets GSE60993 and GSE66360. (B) Removing batch effects between MI datasets. (C)A significant batch effect between the MI combined data. (D) Gene expression profiling data with consistent expression levels after treatment. (E) Merging of the IS datasets GSE22255和GSE16561. (F) Removing batch effects between IS datasets. (G)A significant batch effect between the IS combined data. (H) Gene expression profiling data with consistent expression levels after treatment. MI: Myocardial Infarction. IS: Ischemic Stroke.
