# Supplementary material for: Integrative analyses and validation of ferroptosis-related genes and mechanisms associated with cerebrovascular and cardiovascular ischemic diseases
Source: BMC Genomics. 2023 Dec 4;24:731. doi: 10.1186/s12864-023-09829-w (PMC10694919; doi:10.1186/s12864-023-09829-w)
Supplement: Supplementary file 6 — Additional file 6: Table S5. GO enrichment results of MI. [file 12864_2023_9829_MOESM6_ESM.docx]

Table S5. GO enrichment results of MI.

| **Category** | **ID** | **Description** | **pvalue** |
| --- | --- | --- | --- |
| BP | GO:0001659 | temperature homeostasis | 8.69E-12 |
| BP | GO:0032496 | response to lipopolysaccharide | 5.07E-11 |
| BP | GO:0002237 | response to molecule of bacterial origin | 8.40E-11 |
| BP | GO:0048660 | regulation of smooth muscle cell proliferation | 7.01E-10 |
| BP | GO:0048659 | smooth muscle cell proliferation | 8.17E-10 |
| BP | GO:0048661 | positive regulation of smooth muscle cell proliferation | 1.29E-09 |
| BP | GO:0033002 | muscle cell proliferation | 6.53E-09 |
| BP | GO:0106106 | cold-induced thermogenesis | 9.20E-09 |
| BP | GO:0120161 | regulation of cold-induced thermogenesis | 9.20E-09 |
| BP | GO:1990845 | adaptive thermogenesis | 1.54E-08 |
| CC | GO:0031968 | organelle outer membrane | 9.54E-05 |
| CC | GO:0019867 | outer membrane | 9.87E-05 |
| CC | GO:0071682 | endocytic vesicle lumen | 0.00027 |
| CC | GO:0045121 | membrane raft | 0.000402 |
| CC | GO:0098857 | membrane microdomain | 0.000402 |
| CC | GO:0001891 | phagocytic cup | 0.000408 |
| CC | GO:0005741 | mitochondrial outer membrane | 0.00131 |
| CC | GO:1904724 | tertiary granule lumen | 0.00157 |
| CC | GO:0005901 | caveola | 0.00363 |
| CC | GO:0044853 | plasma membrane raft | 0.00680 |
| MF | GO:0046982 | protein heterodimerization activity | 0.000469 |
| MF | GO:0001228 | DNA-binding transcription activator activity, RNA polymerase II-specific | 0.00152 |
| MF | GO:0001216 | DNA-binding transcription activator activity | 0.00160 |
| MF | GO:0016209 | antioxidant activity | 0.00429 |
| MF | GO:0001227 | DNA-binding transcription repressor activity, RNA polymerase II-specific | 0.00505 |
| MF | GO:0001217 | DNA-binding transcription repressor activity | 0.00519 |
| MF | GO:0002020 | protease binding | 0.0102 |
| MF | GO:0020037 | heme binding | 0.0108 |
