# Supplementary material for: Integrative analyses and validation of ferroptosis-related genes and mechanisms associated with cerebrovascular and cardiovascular ischemic diseases
Source: BMC Genomics. 2023 Dec 4;24:731. doi: 10.1186/s12864-023-09829-w (PMC10694919; doi:10.1186/s12864-023-09829-w)
Supplement: Supplementary file 7 — Additional file 7: Table S6. KEGG enrichment results of MI. [file 12864_2023_9829_MOESM7_ESM.docx]

Table S6. KEGG enrichment results of MI.

| Category | ID | Description | pvalue |
| --- | --- | --- | --- |
| KEGG | hsa05140 | Leishmaniasis | 1.23E-05 |
| KEGG | hsa04657 | IL-17 signaling pathway | 2.71E-05 |
| KEGG | hsa04064 | NF-kappa B signaling pathway | 4.03E-05 |
| KEGG | hsa04668 | TNF signaling pathway | 5.40E-05 |
| KEGG | hsa04216 | Ferroptosis | 6.31E-05 |
| KEGG | hsa04217 | Necroptosis | 0.000210 |
| KEGG | hsa05321 | Inflammatory bowel disease | 0.000251 |
| KEGG | hsa04920 | Adipocytokine signaling pathway | 0.000299 |
| KEGG | hsa04621 | NOD-like receptor signaling pathway | 0.000368 |
| KEGG | hsa05133 | Pertussis | 0.000398 |
