# Supplementary material for: Integrative analyses and validation of ferroptosis-related genes and mechanisms associated with cerebrovascular and cardiovascular ischemic diseases
Source: BMC Genomics. 2023 Dec 4;24:731. doi: 10.1186/s12864-023-09829-w (PMC10694919; doi:10.1186/s12864-023-09829-w)
Supplement: Supplementary file 8 — Additional file 8: Table S7. GO enrichment results of IS. [file 12864_2023_9829_MOESM8_ESM.docx]

Table S7. GO enrichment results of IS.

| **Category** | **ID** | **Description** | **pvalue** |
| --- | --- | --- | --- |
| BP | GO:0001659 | temperature homeostasis | 8.59E-09 |
| BP | GO:0072593 | reactive oxygen species metabolic process | 9.21E-09 |
| BP | GO:0031667 | response to nutrient levels | 7.20E-08 |
| BP | GO:1903008 | organelle disassembly | 1.34E-07 |
| BP | GO:0071496 | cellular response to external stimulus | 1.44E-07 |
| BP | GO:0062197 | cellular response to chemical stress | 2.32E-07 |
| BP | GO:0006979 | response to oxidative stress | 3.48E-07 |
| BP | GO:0000422 | autophagy of mitochondrion | 3.66E-07 |
| BP | GO:0061726 | mitochondrion disassembly | 3.66E-07 |
| BP | GO:2001233 | regulation of apoptotic signaling pathway | 3.85E-07 |
| BP | GO:1901653 | cellular response to peptide | 4.16E-07 |
| BP | GO:0055072 | iron ion homeostasis | 4.88E-07 |
| BP | GO:0034599 | cellular response to oxidative stress | 6.40E-07 |
| BP | GO:0106106 | cold-induced thermogenesis | 6.60E-07 |
| BP | GO:0120161 | regulation of cold-induced thermogenesis | 6.60E-07 |
| BP | GO:0016236 | macroautophagy | 6.98E-07 |
| BP | GO:0120162 | positive regulation of cold-induced thermogenesis | 1.07E-06 |
| BP | GO:1990845 | adaptive thermogenesis | 1.18E-06 |
| BP | GO:2000377 | regulation of reactive oxygen species metabolic process | 1.18E-06 |
| BP | GO:0071453 | cellular response to oxygen levels | 2.63E-06 |
| CC | GO:0000421 | autophagosome membrane | 2.26E-07 |
| CC | GO:0005776 | autophagosome | 1.09E-06 |
| CC | GO:0034774 | secretory granule lumen | 1.24E-05 |
| CC | GO:0060205 | cytoplasmic vesicle lumen | 1.33E-05 |
| CC | GO:0031983 | vesicle lumen | 1.39E-05 |
| CC | GO:0035580 | specific granule lumen | 5.70E-05 |
| CC | GO:0000407 | phagophore assembly site | 0.000186 |
| CC | GO:0005685 | U1 snRNP | 0.000186 |
