# Supplementary material for: Integrative analyses and validation of ferroptosis-related genes and mechanisms associated with cerebrovascular and cardiovascular ischemic diseases
Source: BMC Genomics. 2023 Dec 4;24:731. doi: 10.1186/s12864-023-09829-w (PMC10694919; doi:10.1186/s12864-023-09829-w)
Supplement: Supplementary file 9 — Additional file 9: Table S8. KEGG enrichment results of IS. [file 12864_2023_9829_MOESM9_ESM.docx]

Table S8. KEGG enrichment results of IS.

| Category | ID | Description | pvalue |
| --- | --- | --- | --- |
| KEGG | hsa04140 | Autophagy - animal | 9.78E-07 |
| KEGG | hsa04216 | Ferroptosis | 2.94E-06 |
| KEGG | hsa04137 | Mitophagy - animal | 0.000687 |
| KEGG | hsa05235 | PD-L1 expression and PD-1 checkpoint pathway in cancer | 0.00152 |
| KEGG | hsa04657 | IL-17 signaling pathway | 0.00186 |
| KEGG | hsa00270 | Cysteine and methionine metabolism | 0.00289 |
| KEGG | hsa04931 | Insulin resistance | 0.00309 |
| KEGG | hsa04066 | HIF-1 signaling pathway | 0.00319 |
| KEGG | hsa05167 | Kaposi sarcoma-associated herpesvirus infection | 0.00448 |
| KEGG | hsa00590 | Arachidonic acid metabolism | 0.00480 |
