# Supplementary material for: Integrative analyses and validation of ferroptosis-related genes and mechanisms associated with cerebrovascular and cardiovascular ischemic diseases
Source: BMC Genomics. 2023 Dec 4;24:731. doi: 10.1186/s12864-023-09829-w (PMC10694919; doi:10.1186/s12864-023-09829-w)
Supplement: Supplementary file 10 — Additional file 10: Table S9. GSEA-MI. [file 12864_2023_9829_MOESM10_ESM.docx]

Table S9. GSEA-MI.

| ID | ES | NES | pvalue |
| --- | --- | --- | --- |
| go acute inflammatory response | 0.685 | 2.48 | 1.00E-10 |
| go cellular response to biotic stimulus | 0.646 | 2.59 | 1.00E-10 |
| go cellular response to external stimulus | 0.530 | 2.21 | 1.00E-10 |
| go cellular response to lipid | 0.456 | 1.99 | 1.00E-10 |
| go cellular response to molecule of bacterial origin | 0.649 | 2.55 | 1.00E-10 |
| go cell chemotaxis | 0.566 | 2.35 | 1.00E-10 |
| go collagen containing extracellular matrix | 0.484 | 2.08 | 1.00E-10 |
| go cytokine activity | 0.547 | 2.20 | 1.00E-10 |
| go cytokine secretion | 0.673 | 2.45 | 1.00E-10 |
| go defense response to bacterium | 0.582 | 2.35 | 1.00E-10 |
| go extracellular matrix | 0.471 | 2.05 | 1.00E-10 |
| go granulocyte chemotaxis | 0.683 | 2.50 | 1.00E-10 |
| go granulocyte migration | 0.666 | 2.51 | 1.00E-10 |
| go large ribosomal subunit | -0.603 | -2.36 | 1.00E-10 |
| go leukocyte chemotaxis | 0.598 | 2.39 | 1.00E-10 |
| go leukocyte migration | 0.519 | 2.24 | 1.00E-10 |
| go mitochondrial gene expression | -0.622 | -2.53 | 1.00E-10 |
| go mitochondrial matrix | -0.511 | -2.37 | 1.00E-10 |
| go mitochondrial protein complex | -0.528 | -2.29 | 1.00E-10 |
| go mitochondrial translation | -0.634 | -2.44 | 1.00E-10 |
