# Supplementary material for: Integrative analyses and validation of ferroptosis-related genes and mechanisms associated with cerebrovascular and cardiovascular ischemic diseases
Source: BMC Genomics. 2023 Dec 4;24:731. doi: 10.1186/s12864-023-09829-w (PMC10694919; doi:10.1186/s12864-023-09829-w)
Supplement: Supplementary file 11 — Additional file 11: Table S10. GSEA-IS. [file 12864_2023_9829_MOESM11_ESM.docx]

Table S10. GSEA-IS.

| ID | ES | NES | pvalue |
| --- | --- | --- | --- |
| go azurophil granule | 0.628 | 2.34 | 1.00E-10 |
| go catalytic activity acting on rna | -0.524 | -2.01 | 1.00E-10 |
| go cellular response to biotic stimulus | 0.565 | 2.22 | 1.00E-10 |
| go cellular response to external stimulus | 0.511 | 2.08 | 1.00E-10 |
| go cellular response to molecule of bacterial origin | 0.571 | 2.23 | 1.00E-10 |
| go coagulation | 0.491 | 2.00 | 1.00E-10 |
| go large ribosomal subunit | -0.677 | -2.32 | 1.00E-10 |
| go mitochondrial gene expression | -0.744 | -2.66 | 1.00E-10 |
| go mitochondrial large ribosomal subunit | -0.76 | -2.38 | 1.00E-10 |
| go mitochondrial matrix | -0.587 | -2.37 | 1.00E-10 |
| go mitochondrial protein complex | -0.725 | -2.71 | 1.00E-10 |
| go mitochondrial translation | -0.752 | -2.65 | 1.00E-10 |
| go mitochondrial translational termination | -0.778 | -2.63 | 1.00E-10 |
| go myeloid leukocyte mediated immunity | 0.619 | 2.66 | 1.00E-10 |
| go ncrna metabolic process | -0.594 | -2.32 | 1.00E-10 |
| go ncrna processing | -0.625 | -2.39 | 1.00E-10 |
| go organellar ribosome | -0.780 | -2.62 | 1.00E-10 |
| go organelle inner membrane | -0.539 | -2.18 | 1.00E-10 |
| go peptide biosynthetic process | -0.464 | -1.90 | 1.00E-10 |
| go platelet alpha granule | 0.688 | 2.38 | 1.00E-10 |
