# Supplementary material for: Integrative analyses and validation of ferroptosis-related genes and mechanisms associated with cerebrovascular and cardiovascular ischemic diseases
Source: BMC Genomics. 2023 Dec 4;24:731. doi: 10.1186/s12864-023-09829-w (PMC10694919; doi:10.1186/s12864-023-09829-w)
Supplement: Supplementary file 12 — Additional file 12: Table S11. GSVA-MI. [file 12864_2023_9829_MOESM12_ESM.docx]

Table S11. GSVA-MI.

| hallmark | gene | correlation | P value |
| --- | --- | --- | --- |
| hallmark tnfa signaling via nfkb | ACSL1 | 0.673 | 5.60E-10 |
| hallmark hypoxia | ACSL1 | 0.483 | 3.84E-05 |
| hallmark cholesterol homeostasis | ACSL1 | 0.544 | 2.28E-06 |
| hallmark mitotic spindle | ACSL1 | 0.260 | 0.0348 |
| hallmark wnt beta catenin signaling | ACSL1 | 0.138 | 0.267 |
| hallmark tgf beta signaling | ACSL1 | 0.469 | 6.91E-05 |
| hallmark il6 jak stat3 signaling | ACSL1 | 0.572 | 5.21E-07 |
| hallmark dna repair | ACSL1 | 0.179 | 0.149 |
| hallmark g2m checkpoint | ACSL1 | 0.109 | 0.383 |
| hallmark apoptosis | ACSL1 | 0.493 | 2.53E-05 |
| hallmark notch signaling | ACSL1 | 0.337 | 0.00553 |
| hallmark adipogenesis | ACSL1 | 0.407 | 0.000676 |
| hallmark estrogen response early | ACSL1 | -0.0117 | 0.925 |
| hallmark estrogen response late | ACSL1 | 0.0306 | 0.807 |
| hallmark androgen response | ACSL1 | 0.492 | 2.71E-05 |
| hallmark myogenesis | ACSL1 | -0.0440 | 0.725 |
| hallmark protein secretion | ACSL1 | 0.351 | 0.00378 |
| hallmark interferon alpha response | ACSL1 | 0.292 | 0.0169 |
| hallmark interferon gamma response | ACSL1 | 0.371 | 0.00211 |
| hallmark tnfa signaling via nfkb | ACSL1 | 0.673 | 5.60E-10 |
