# Supplementary material for: Integrative analyses and validation of ferroptosis-related genes and mechanisms associated with cerebrovascular and cardiovascular ischemic diseases
Source: BMC Genomics. 2023 Dec 4;24:731. doi: 10.1186/s12864-023-09829-w (PMC10694919; doi:10.1186/s12864-023-09829-w)
Supplement: Supplementary file 13 — Additional file 13: Table S12. GSVA-IS. [file 12864_2023_9829_MOESM13_ESM.docx]

Table S12. GSVA-IS.

| hallmark | gene | correlation | P value |
| --- | --- | --- | --- |
| hallmark tnfa signaling via nfkb | ACSL1 | 0.677 | 3.82E-09 |
| hallmark hypoxia | ACSL1 | 0.511 | 3.48E-05 |
| hallmark cholesterol homeostasis | ACSL1 | 0.532 | 1.42E-05 |
| hallmark mitotic spindle | ACSL1 | 0.214 | 0.102 |
| hallmark wnt beta catenin signaling | ACSL1 | 0.152 | 0.250 |
| hallmark tgf beta signaling | ACSL1 | -0.0858 | 0.517 |
| hallmark il6 jak stat3 signaling | ACSL1 | 0.488 | 8.73E-05 |
| hallmark dna repair | ACSL1 | 0.118 | 0.369 |
| hallmark g2m checkpoint | ACSL1 | 0.290 | 0.0257 |
| hallmark apoptosis | ACSL1 | 0.483 | 0.000103 |
| hallmark notch signaling | ACSL1 | 0.219 | 0.095 |
| hallmark adipogenesis | ACSL1 | 0.181 | 0.169 |
| hallmark estrogen response early | ACSL1 | 0.0938 | 0.479 |
| hallmark estrogen response late | ACSL1 | 0.109 | 0.410 |
| hallmark androgen response | ACSL1 | 0.520 | 2.41E-05 |
| hallmark myogenesis | ACSL1 | -0.194 | 0.139 |
| hallmark protein secretion | ACSL1 | 0.159 | 0.228 |
| hallmark interferon alpha response | ACSL1 | 0.0736 | 0.579 |
| hallmark interferon gamma response | ACSL1 | 0.212 | 0.106 |
| hallmark apical junction | ACSL1 | -0.245 | 0.0609 |
