# Supplementary material for: Integrative analyses and validation of ferroptosis-related genes and mechanisms associated with cerebrovascular and cardiovascular ischemic diseases
Source: BMC Genomics. 2023 Dec 4;24:731. doi: 10.1186/s12864-023-09829-w (PMC10694919; doi:10.1186/s12864-023-09829-w)
Supplement: Supplementary file 14 — Additional file 14: Fig. S2. Construction of co-expression modules for MI and IS. (A) The optimal soft threshold for MI gene module scale-free R is 0.80 to ensure a non-scaling topology. (B) Plotting the scatter plot of gray module gene significance vs. module membership for MI. (C) Plotting the scatter plot of yellow module gene significance vs. module membership for MI. (D)The optimal soft threshold for IS gene module scale-free R is 0.80 to ensure a non-scaling topology. (E) Plotting the scatter plot of pink module gene significance vs. module membership for IS. (F) Plotting the scatter plot of blue module gene significance vs. module membership for IS. MI: Myocardial Infarction. IS: Ischemic Stroke. [file 12864_2023_9829_MOESM14_ESM.zip › Additional file 14 Figure S2.docx]

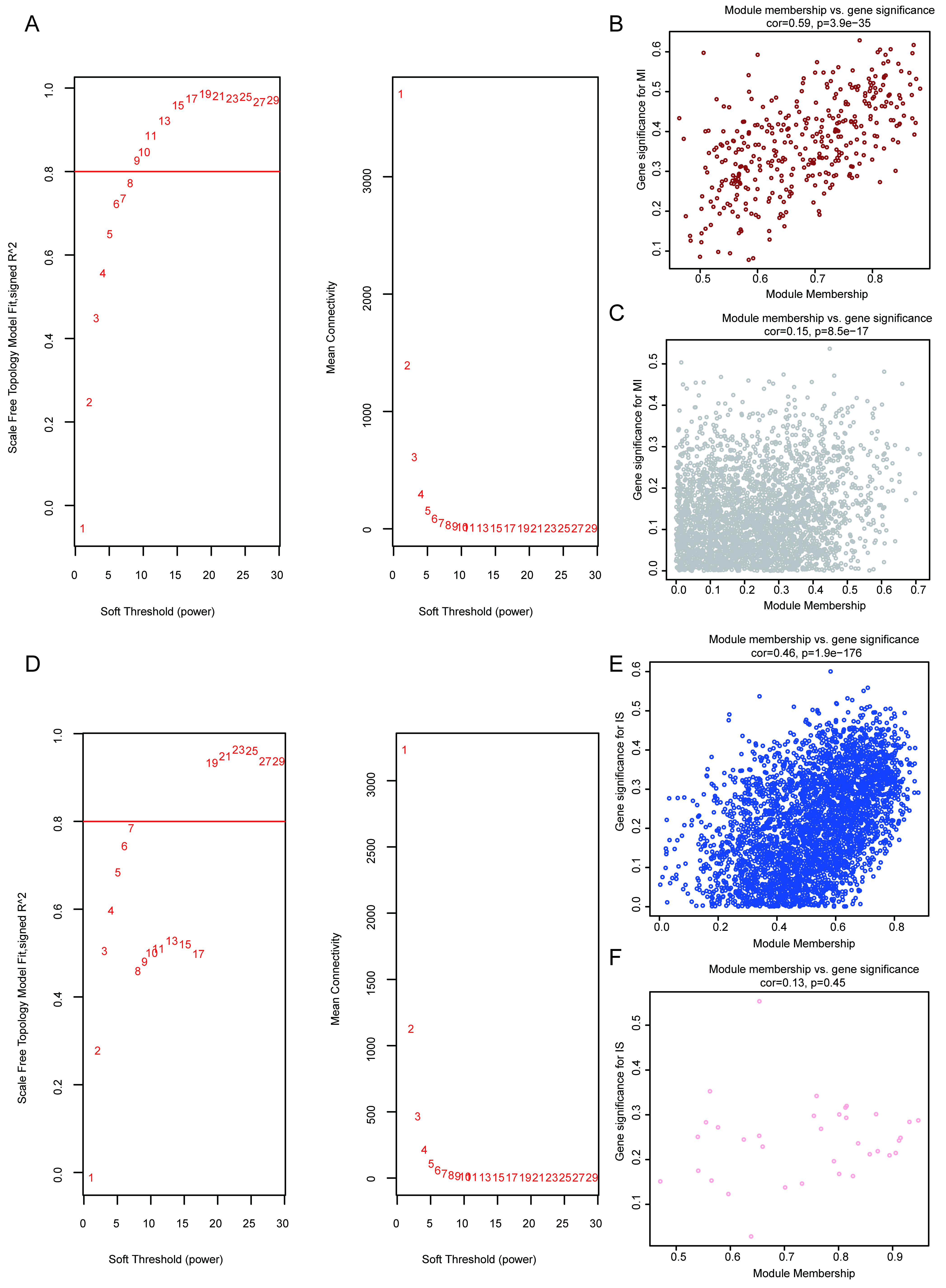


**Fig S2. Construction of co-expression modules for MI and IS.** (A) The optimal soft threshold for MI gene module scale-free R is 0.80 to ensure a non-scaling topology. (B) Plotting the scatter plot of gray module gene significance vs. module membership for MI. (C) Plotting the scatter plot of yellow module gene significance vs. module membership for MI. (D)The optimal soft threshold for IS gene module scale-free R is 0.80 to ensure a non-scaling topology. (E) Plotting the scatter plot of pink module gene significance vs. module membership for IS. (F) Plotting the scatter plot of blue module gene significance vs. module membership for IS. MI: Myocardial Infarction. IS: Ischemic Stroke.
